# Performance of longitudinal item response theory models in shortened or partial assessments

**DOI:** 10.1007/s10928-020-09697-x

**Published:** 2020-07-02

**Authors:** Leticia Arrington, Sebastian Ueckert, Malidi Ahamadi, Sreeraj Macha, Mats O. Karlsson

**Affiliations:** 1 Department of Pharmaceutical Biosciences, Uppsala University, P.O. Box 591, 751 24 Uppsala, Sweden; 2 Merck & Co. Inc, Kenilworth, NJ USA

**Keywords:** Item response theory, Composite score, Pharmacometrics, Item information

## Abstract

This work evaluates the performance of longitudinal item response (IR) theory models in shortened assessments using an existing model for part II and III of the MDS-UPDRS score. Based on the item information content, the assessment was reduced by removal of items in multiple increments and the models’ ability to recover the item characteristics of the remaining items at each level was evaluated. This evaluation was done for both simulated and real data. The metric of comparison in both cases was the item information function. For real data, the impact of shortening on the estimated disease progression and drug effect was also studied. In the simulated data setting, the item characteristics did not differ between the full and the shortened assessments down to the lowest level of information remaining; indicating a considerable independence between items. In contrast when reducing the assessment in a real data setting, a substantial change in item information was observed for some of the items. Disease progression and drug effect estimates also decreased in the reduced assessments. These changes indicate a shift in the measured construct of the shortened assessment and warrant caution when comparing results from a partial assessment with results from the full assessment.

**Electronic supplementary material:**

The online version of this article (10.1007/s10928-020-09697-x) contains supplementary material, which is available to authorized users.

## Introduction

Composite scores from clinical assessments which use rating scales are a common method for the evaluation of ability or disability in a wide range of therapeutic areas. Generally, they aim at capturing different aspects of a disease by combining a variety of symptoms into a single composite score. These scores are used in clinical practice for diagnosis and to guide treatment of patients, but are also common endpoints in clinical trials. The statistical data analysis of clinical trials with composite assessments as endpoints is traditionally based on the total composite score only. Similarly, pharmacometric models of disease progression historically described the evolution of disease with total score [1–3]. In recent years, however, item response (IR) theory has gained increased interest in application within pharmacometric frameworks [4]. IR originated in the field of psychometrics where it is a common methodology for the evaluation of achievement test outcomes. IR analysis is a statistical methodology used in the interpretation of clinical assessments that seeks to establish a relationship between the single underlying hidden latent variable, the concept we wish to measure (e.g. cognitive ability or disability), and the item (i.e. questions) responses in a measure [5]. The latent variable is not directly observed and can be viewed as an abstract concept. IR acknowledges the composite nature of the score by describing the response for each item and, hence, is also well adapted to studying shortened and reduced assessments. The interest for such shortened assessments has been increasing over the past years both in clinical practice [6] and demonstrated by Younis et al. (Food and Drug Administration) in their evaluation of modified positive and negative syndrome scale (PANSS) in schizophrenia in 2018. As well as in academia with multiple publications pointing to the possibility of decreasing assessment burden without significant loss of information [7, 8]. This work is concerned with the consequences of such reductions, both in terms of measured construct as well as in regards to the stability of the pharmacometric model.

In its simplest form, an IR model assumes that the probability for a response to an item can be described through an item characteristic function (ICF) which depends on item-specific parameter values and a subject-specific latent variable that is shared across items. Pharmacometric IR models use the same concepts, but in addition try to capture how the latent variable changes over time and under treatment. There are many examples where the application of the IR methodology in pharmacometrics has been utilized to better understand disease progression characteristics of a disease using different scale types; e.g., ADAS-COG score in Alzheimer’s disease (AD) [7], MDS-UPDRS (Movement Disorder Society Unified Parkinson’s Disease Rating Scale) in Parkinson’s Disease [9, 10], and Kurtzke Expanded Disability Status Scale (EDSS) score in Multiple Sclerosis [8]. Longitudinal IR models have been shown to provide higher power to detect a drug effect compared to analyses based on the total score only (assuming adequate model fit) [7, 8, 10]. In these pharmacometric IR models 20–40% fewer subjects were required to achieve 80% power compared to total score [7, 11, 12].

All parametric modelling approaches implicitly assume a weighting of the data used during parameter estimation and Fisher information quantifies this weighting. The Cramer-Rao bound also establishes an explicit link between Fisher information and the maximal attainable precision of a parameter [13, 14]. Within the IR framework, Fisher information can be calculated on the item level as a function of the latent variable and reveal how much data for each item is contributing in a particular disability or disease severity group. This framework is therefore well suited in comparing the information content of different assessments. An assessment with twice the Fisher information requires only half as many subjects to achieve the same parameter precision, giving this seemingly abstract quantity a very relatable meaning. It is therefore not surprising, that the quantification of information of the individual assessment components is a popular topic in the pharmacometric application of IR. Often, authors find that the majority of the information is contained in a fraction of the total number of items [7, 8]. In addition to reducing assessment burden, subsets of an assessment can also be of interest for other reasons, such as clinical relevance to specific component of the disease, reduction of noise contributed by less informative items or anticipated target of drug effect. Provided the increased development effort of a longitudinal IR model compared to a total score model, utilizing only part of the assessment might also be appealing.

The behavior of longitudinal IR models in shortened assessments however, has not been sufficiently studied. Few studies that evaluate the proposed shortened assessments typically do so in a simulation setting only [8]. It therefore remains largely unclear how these shorter assessments perform in terms of estimation and in comparison to a model for the total assessment. The aim of this work was to help understand these aspects.

The dimensionality (i.e., number of latent variables or dimensions) of the data depends on both the assessment and the subject sample [15]. Components of a well designed assessment are considered unidimensional when a single latent variable dominates the influence of the probability of a subjects’ response to a series of items (i.e. motor score). When a unidimensional IR model is applied to data from an assessment that may be capturing more than one latent trait, which is often the case with real data, the model estimates reflect a composite or average measure of the multiple latent variables [16]. Simultaneously, our work can also yield insights into the behavior of IR models for clinical assessments with few items.

The real world clinical data for this work comes from the observational Parkinson’s Progression Markers Initiative (PPMI) study. The PPMI study evaluated the level of disability of the participants using the MDS-UPDRS, a revision of the UPDRS scale, designed to detect smaller changes in disease in patients with early or milder impairment. Our study is based on the work of Buatois et al. [10] which developed a longitudinal IR model for parts I to III of the MDS-UPDRS scale [17] data from the PPMI study. Here, we utilize both the real clinical trial data and the model component for part II and III, the motor item subscale, only. In this work, we will focus on methodologic questions and avoid clinical interpretations.

Our overall working scheme was to gradually reduce the number of items from the full assessment scale and evaluate the impact on different model components, first the item response component then the latent variable model. We start our evaluation using simulated data and then proceed to using real data. In our workflow, Fisher information was of special importance. It was used both as means of reducing the assessment in a directed manner as well as a metric to evaluate the impact of the shortening.

## Methods

### Data

Data used for this evaluation were obtained from the Parkinson’s Progression Markers Initiative (PPMI) database (www.ppmi-info.org/data) version available as of February 2015. PPMI is an observational study sponsored and partially funded by The Michael J. Fox Foundation for Parkinson’s Research (MJFF). Only the de novo PD patient’s data was used for analysis. The de novo PD patients were stage I or II based on Hoehn & Yahr stage classification and had not used PD medication for more than 60 days prior to baseline [10]. This resulted in N = 423 de novo PD patients where each patient contributed to each item’s assessment measurement across 10 visits up to trial month 48.

Only the motor sub-scale was used for analysis, thus reducing the number of items for evaluation from 65 to 34. Many aspects regarding data handling were retained from the previously published analysis; Item 3.11 freezing gait was excluded during analysis because greater than 98% of the responses were zero, items with repeat measurement assessments were included in the analysis and data for response categories were consolidated when less than 2.5% of the subjects had responses in the highest or more severe categories for specific items [10]. Items were renumbered from their MDS-UPDRS item classification numbers to 1–65 starting from Part I.

### Longitudinal IR model

The motor subcomponent of the MDS-UPDRS unidimensional (i.e., using a single latent variable) IR model developed by Buatois et al. was used in this work [10]. This model described the PPMI data well as demonstrated by simulation based diagnostics [10]. The IR model was developed using assessments prior to subjects beginning treatment or at time points when the assessment was completed pre-dose. For subject $i$ and item $j$, the graded response model describes the probability of achieving a response of at least s at time point ${t}_{k}$ as

1 $$P\left( {Y_{ijk} \ge s|D_{i} \left( {t_{k} } \right)} \right) = \frac{{e^{{a_{j} \left( {D_{i} \left( {t_{k} } \right) - b_{j,s} } \right)}} }}{{1 + e^{{a_{j} \left( {D_{i} \left( {t_{k} } \right) - b_{j,s} } \right)}} }}$$

and, consequentially, to achieve a score of exactly $s$ as

2 $$P\left( {Y_{ijk} = s|D_{i} \left( {t_{k} } \right)} \right) = P\left( {Y_{ijk} \ge s|D_{i} \left( {t_{k} } \right)} \right) - P\left( {Y_{ijk} \ge s + 1|D_{i} \left( {t_{k} } \right)} \right)$$

where ${D}_{i}({t}_{k})$ is the subject’s time-dependent latent variable value, ${a}_{j}$ is the item-specific discrimination parameter and ${b}_{j,s}$ is the threshold parameter for that specific item and score. The evolution of the latent variable over time was evaluated with models with and without treatment effect;

3 $$D_{i} \left( {t_{k} } \right) = D_{i}^{0} + \alpha_{i} t_{k}$$

4 $$D_{i} \left( {t_{k} } \right) = D_{i}^{0} + \alpha_{i} t_{k} + E_{i}^{0}$$

where ${\mathrm{D}}_{\mathrm{i}}^{0}$ is the baseline latent variable value, ${\alpha }_{i}$ is the disease progression rate and ${E}_{i}^{0}$ is the latent variable offset for the symptomatic drug effect (assumed 0 when subjects were not treated). All three parameters were assumed to follow a normal distribution in the population on the logit scale.

Representative diagnostic plots for ICC and model visual predictive checks (VPC) to demonstrate adequacy of model fit are presented in Supplementary Online Resource 2.

### Item information

For each item *j* the item information function was calculated as minus the expectation of the second derivative of the log-likelihood [18], i.e.,

5 $$I_{j} \left( {D_{i} } \right) = - \mathop \sum \limits_{s = 0}^{{s_{j} }} P\left( {Y_{ij} = s|D_{i} } \right)\frac{{\partial^{2} \log P\left( {Y_{ij} = s|D_{i} } \right)}}{{\partial D_{i}^{2} }}$$

where $P\left({Y}_{ij}=s|{D}_{i}\right)$ is the response (s) probability for the disability ${D}_{i}$ as defined above. Furthermore, the population information,${\text{I}}_{j}$ was defined as the item information integrated over the entire disability range, i.e.,

6 $$I_{j} = \int\limits_{{ - \infty }}^{\infty } {p\left( {D_{i} } \right)I_{j} \left( {D_{i} } \right)d D_{i} }$$

where $p\left({D}_{i}\right)$ is the probability density of the latent variable distribution in the population.

### Workflow

All model parameters in the longitudinal IR model, described above, were simultaneously estimated from the data. This setting, with all items present, will be referred to as the “100%” scenario moving forward. Items were then ranked in order of calculated population Fisher information content from most informative to least (Table 1). In the subsequent steps, subsets of the data with fewer items were analyzed using the same model. The item reduction proceeded in approximately 10% information content decrements, removed from the lower end and as a sensitivity analysis the upper end of the item information ranking.

**Table 1:** Item level ranking of MDS-UPDRS components by information content and total cumulative % information content for items on motor subscale (34 Items)

			Cumulative % of total information remaining	
Item	Test name	Information at baseline for latent variable	Removal from most informative direction	Removal from least informative direction
49	Global Spont. of movement	0.58	100	
35	Finger Tap-left hand	0.57		
37	Hand Move-left hand	0.50	81	
18	Dressing	0.42		19
39	Pronation-supine-left hand	0.42	71	
31	Rigidity_LUE	0.41		29
28	Facial expression	0.37		
29	Rigidity_Neck	0.33	59	
36	Hand move-right hand	0.32		41
34	Finger Tap-right hand	0.32	51	
41	Toe tap-left foot	0.32		49
24	Getting out of bed	0.30		
33	Rigidity_LLE	0.29	41	
43	Leg agility-Left leg	0.29		59
42	Leg agility-Right leg	0.27		
40	Toe tap-right foot	0.24		
48	Posture	0.21	29	
27	Speech 3.1	0.21		71
32	Rigidity_RLE	0.21		
17	Eating tasks	0.20		
25	Walking and balancing	0.20	20	
38	Pronation-supine-right hand	0.19		80
30	Rigidity_RUE	0.19		
21	Doing hobbies and other activities	0.19		
22	Turning in bed	0.16		
19	Hygiene	0.16	10	
14	Speech	0.15		90
45	Gait	0.14		
15	Saliva and drooling	0.14		
44	Arising from chair	0.13		
20	Handwriting	0.10		
26	Freezing	0.09		
16	Chewing and swallowing	0.08		
47	Postural stability	0.04		100

Items on row with percentage are also included in the information content decrement step

The behavior of the reduced-item analyses were evaluated for both the item response model and the latent variable model. The former was judged by graphically comparing the item level efficiency calculated as the ratio of the item information function for the reduced and the 100% scenario. For the latent variable model, stability was assessed by comparing the parameter estimates for the mean slope and drug effect of the reduced-item analyses and the 100% scenario.

The evaluation was done for real data as well as using data simulated from the 100% scenario model.

Implementation and parameter estimation of the above-described model was performed using Nonlinear mixed effect model software (NONMEM) version 7.4.2. All parameters were jointly estimated using the Laplacian (second order conditional) estimation method.

## Results

### Item information

**Fig. 1:**
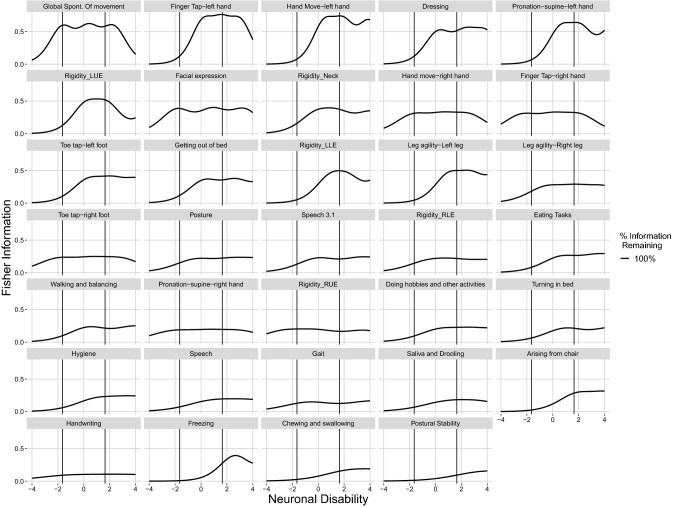
Item information curves for MDS-UPDRS motor items versus Disability for 100% scenario *Vertical Lines* indicate the disability range for 95% of the reference population

The item information curves as a function of disability as estimated under the 100% scenario are represented in Fig. 1. In general, an item information curve visualizes the importance of observations from a subject with a particular disability level for the estimation of the latent variable parameters. The overall importance of an item can be gauged from the amplitude of the item information curve, whereas the importance of the item in different disability populations is reflected in the location of maxima and minima within an item information curve. There are some items that are more informative for the center of the population (i.e. item 49 “Global Spontaneity of Movement”) and others that are more informative in the lower or higher disability populations or the tails of the disability continuum.

The ranking of items by their population information content is presented in Table 1. Item 49 “Global Spontaneity of Movement” was found to be the most informative item and item 47 “Postural Stability” the least informative. The ratio in information content between the most and least informative item is > 10. Furthermore, the top 10 most informative items were found to contain more than 50% of the total information. There is a generous distribution of information across the 34 items; however, it appears that in each 10% subset there are several items with equal levels of information.

Table 1 also presents the cumulative information in the reduced-item scenarios as well as the inclusion of the items in the different scenarios. The minimum number of items remaining was 3 items (approximately 20% information remaining) when removed from the least informative item direction and 8 items (approximately 10% information remaining) when removed from the most informative item direction.

### Performance for simulated data

The estimation of the item parameters under the reduced information scenarios showed only minor differences when using simulated data. The efficiency, i.e., the ratio of the Fisher information for the reduced and full scenario, was found to be close to 100% for all items (Online Resource 3). Even for the most extreme scenario with only 10% percent of the initial information content, differences in efficiency were less than 10% (for the disability range between − 2 and 2).

**Fig. 2:**
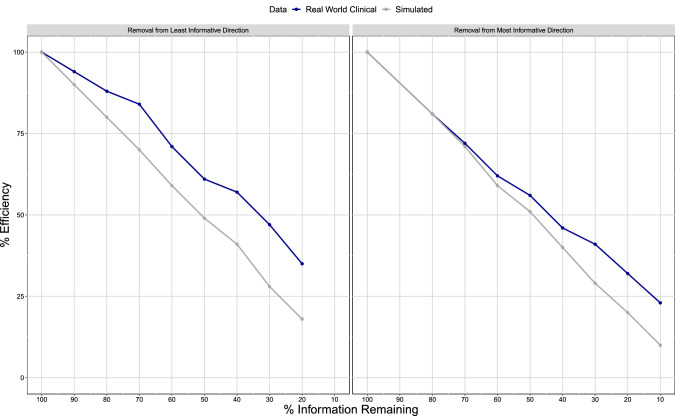
Efficiency on the population level for Real and Simulated data at each level of Reduced Information Content scenarios

This result indicates that the estimation of the item parameters for a particular item was largely unaffected by the presence or absence of other items in the assessment. Figure 2 reaffirms this finding. The figure shows the decrease in efficiency when removing items from the assessment for both real and simulated data. For simulated data, the line closely aligns with the line of identity, i.e., a reduction in the information content yields a drop in efficiency of approximately the same value.

### Performance for real data

**Fig. 3:**
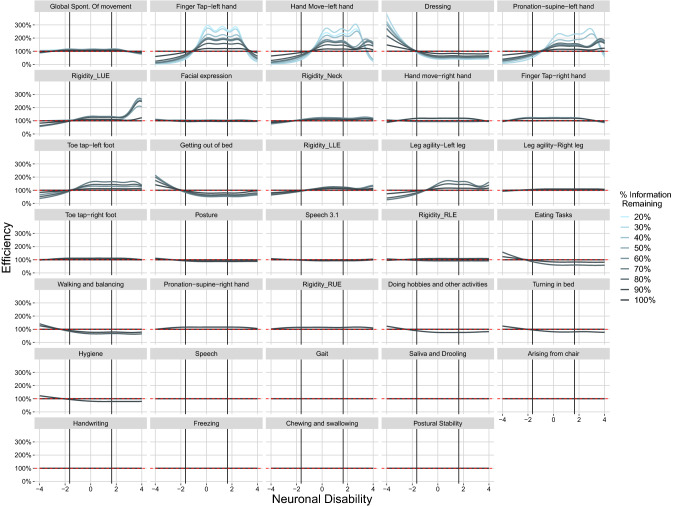
Real world clinical data item level efficiency for MDS-UPDRS motor items versus disability (Removal of Least informative Items First). *Vertical Lines* indicate the disability range for 95% of the reference population

**Fig. 4:**
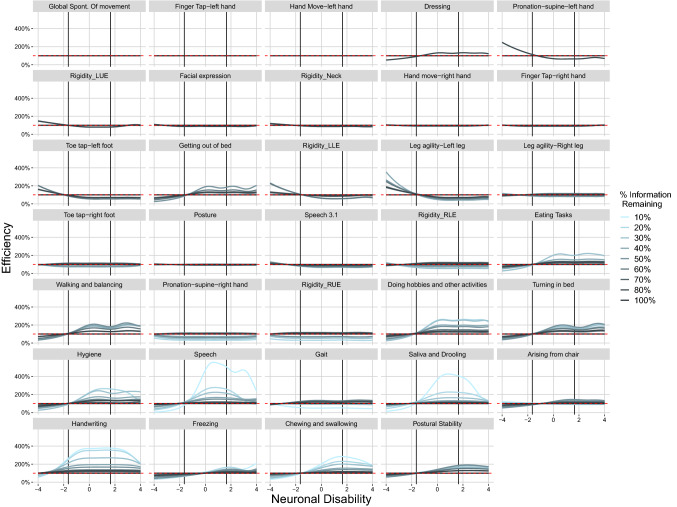
Real world clinical data item level efficiency for MDS-UPDRS motor items versus disability (Removal of Most informative Items First). *Vertical Lines* indicate the disability range for 95% of the reference population

For real data, the impact of removing items from the assessment on the information content of the remaining items is shown in Fig. 3, for the removal of items from the lower end of the ranking, and Fig. 4, for the removal from the upper end. Both figures display efficiency, an item with the same information profile under the reduced and the 100% scenario will, therefore, show as a straight line at 100%. It is evident in both figures that the information profile for some of the items is changing. In general, differences seem to be larger when removing the most informative items first (Fig. 4). It also appears that items with considerable information for the higher disability populations (disability > 2) are more affected by a reduction in the total information than items that are most informative for the center of the population (− 2 < disability < 2). An example for the latter can be seen by comparing item 49 “Global Spontaneity of Movement” and item 35 “Finger Tap-left hand” which shows an increase in efficiency upwards to 300% at the lowest total information level remaining in Fig. 3.

A general trend for items that do exhibit change is the increase in efficiency when the total information is reduced. This can be seen from the increasing amplitude of the information efficiency curves with decreasing information content of the analysis dataset. However, what is seen in many cases is that when there is an increase in information efficiency for a portion of the population there is a decrease observed in the opposite extreme end of the disability population. The effect is generally small when the percentage of removed information content is small; however below approximately 60% information content remaining the increase in relative importance of an item can be substantial. An example is item 35 “Finger Tap-left hand” in Fig. 3. The efficiency increases with every reduction in overall information content, up to a value of larger than 200% relative to the 100% scenario.

In terms of efficiency it is also worth noting that in cases where an item is not initially informative in a portion of the population (Fig. 1) large changes observed in efficiency are based on the small magnitude of information contributed.

Within an item, the relative importance of specific disability populations of subjects is rather stable with the reduction of information content. As an example, one can consider again item 35 “Finger Tap-left hand” in Fig. 3. Most of the information comes from subjects within the disability range of 0 to 2 independent of the overall information content of the assessment. Some notable exceptions are item 37 “Hand Move-left hand” in Fig. 3 and item 14 “Speech” in Fig. 4. When the total information is large, item 37 efficiency is between 120 and 200% even from subjects with disability values around 4. However, for scenarios with 40% information and less, the contributed information from item 37 at a disability value of 4 is reduced as observed by the efficiency dropping below 100% approaching 20% when items are removed from the least informative direction. Item 14 “Speech” shows the opposite effect when items are removed from the most informative direction, with an increasing contribution for high disability populations when the total information content decreases.

The increase in information content for the majority of the items under the reduced information scenarios is also clearly visible in Fig. 2 when comparing the real data setting with the simulation setting. While for the simulated setting, the drop in efficiency corresponds to the expected loss in information due to the removal of items, for the real data efficiency is always higher than expected. When removing items from the least informative direction, the divergence between the simulated and the real data setting occurs already at the 90% scenario. For the removal from the most informative direction, simulated and real data remain in agreement until the 70% information scenario. This difference is most likely due to the difference in the number of removed items, 8 in the first case (to achieve a drop of 10% when removing from the bottom) and 5 in the second (to achieve a drop of 30% when removing from the top).

The results for the real data highlight that for a particular item the estimation of item parameters is not independent of the presence or absence of other items in the assessment. This behavior is in contrast to the simulation setting. The information content for real data and hence the relative weighting of the data, will readjust when dropping items from an assessment.

### Consequences for the latent variable model

**Fig. 5:**
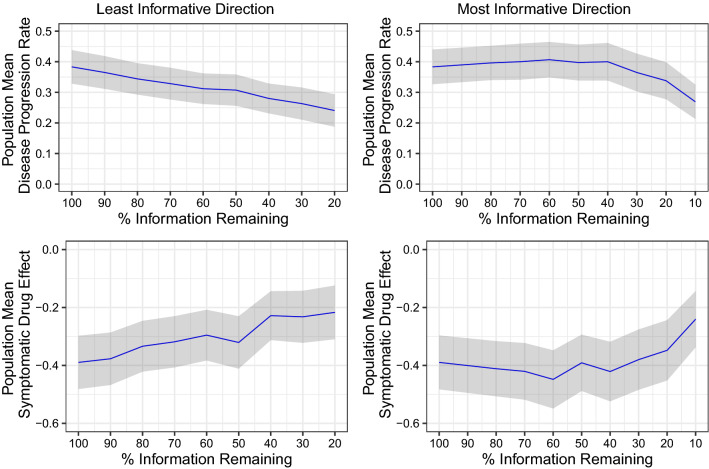
Estimated population mean disease progression rate (latent variable/year) and Symptomatic drug effect estimates and 95% CI (shaded area) for observed motor subscale data at each level of reduced information content with least informative(left panel), Most Informative(right panel) removed first

Estimates for the mean disease progression rate and the mean symptomatic drug effect under the 100% information scenario and when items have been eliminated from the analysis either starting with least informative items (left panels) or with the most informative items (right panels) are represented in Fig. 5. Under both conditions, the overall trend is a decrease of the estimated disease progression rate as well as for the estimated symptomatic drug effect (baseline offset) when total information content is reduced.

Figure 5 also depicts the 95% confidence interval based on the standard error of the estimates. Considering this uncertainty, one could state that when removing the least informative items first, there is no significant difference in mean population parameters until about 60% information content remaining. When removing the most informative items first, one can even go down to 40% remaining information without any significant change.

Tables for the disease progression and drug effect parameter estimates, IIV and relative standard errors (RSE) of each of the models with/without drug effect are presented in the Supplementary Online Resource 1.

The impact of item reduction was also evaluated on individual parameter estimates (results not shown). The empirical Bayes estimates indicate small imprecision around individual estimates for slope as information was reduced compared to the 100% scenario; however, at an item parameter level the model appears to still be robust, with ETA shrinkage of less than 20% at the 100% and 50% information levels and 40% ETA shrinkage at the lowest level of information remaining for both item information removal directions.

## Discussion

In this work, we set out to study the performance of IR models in shortened assessments. We aimed at answering whether with reduced assessments, models can still be reliably estimated and how a reduced assessment model compares to the one developed for the full assessment. The answer to the first questions is a clear “yes”, the answer to the second one is more complex.

Regarding the stability of the model, it is very clear from the results obtained with the simulated data that the removal of items from the assessment has very little impact on the estimation of the ICCs for the remaining items. Our results show that when going from a large 34 item assessment to an assessment with only 4 items (20% information remaining when removing from the lower end), the characteristics of the 4 most informative items can still be reliably estimated. These are encouraging findings for the applicability of IR modeling to small assessments.

Our results also show that for real data it is more complicated, with a few notable consequences of shortening assessments: (i) the increase in item information content for some of the remaining items compared to others, (ii) the increase or decrease in information content for a specific level of disability, and (iii) the observed change in mean values for the latent variable parameters. These outcomes are caused by two non-trivial IR phenomena that are important to be aware of when considering analyzing a subset of an assessment.

First, dropping items that provide a considerable amount of information in the tails of the disability distribution makes it harder to estimate the latent variable value of subjects that are located at these tails. This is most likely why item 37 “Hand Move-left hand” is not informative any longer for subject with a disability value of 4 after the total information content decreases below 50% (Fig. 3). Above 50%, a sufficient number of items in the assessment provide information for high disability values to pinpoint subjects even around a value of 4. Once most of these items are removed from the assessment, however, these extreme subjects shrink toward the center of the distribution and the threshold (i.e. location) parameters larger than 2 cannot be estimated precisely any longer. It might also explain why the disease progression rate estimates decrease with an increasing number of dropped items. When all items are present in the assessment, subjects can be tracked accurately while progressing along the disability continuum. However, this is not true any longer when items with sensitivity at higher disabilities are removed. Once subjects reach a certain disability it appears as if they do not progress any longer but in reality they just cannot be located beyond that disability. This apparent lack of progression is then reflected in a decreased progression rate and a reduction in drug effect. As items are removed the ability to identify a drug effect continues to diminish. Naturally, it is also possible that some items may have been more sensitive to drug effect.

The second phenomenon explains the increase in information content for some items when other items are removed. For clinical assessments of complex conditions, assessments will generally measure a combination of factors or measure along multiple dimensions of a hypothetical symptom space. Each item will measure a slightly different combination of symptoms. When an IR model with one latent variable is used to describe the data (i.e., a unidimensional IR model), the meaning of the latent variable, i.e., what it represents, will be a weighted average of what all items measure. Item information in this situation represents how well each item measures along this average construct. The dominant dimension is measured by all items, but the secondary dimensions may only be influenced by a subset of items [19]. Changing the assessment might change the direction of the average construct and, hence, the information content of the items. In the 100% scenario above, the average construct is a compromise of all items. When few items are left in the assessment, there is less need for compromise and the average construct can align better with some of the remaining items. In consequence, the information content of these items increases. From a practical perspective, however, one should note that the reduced assessment actually measures a slightly different construct than under the 100% scenario. Therefore one needs to be cautious when comparing analysis results utilizing different item subsets.

Our results were obtained for a specific dataset of a subset of the MDS-UPDRS assessment in Parkinson’s disease. The phenomena identified, however, are general. Dropping items informative in the tails of the distribution will reduce the ability to locate subjects in these tails and will hamper the possibility to track subjects progressing over time. Removing items will also change the measured construct, rendering some items more and others less influential. In summary, there is more to take into account than the information content, even from a purely quantitative perspective, when considering shortening an assessment or analyzing only parts. This is also true if a composite score is used for the analysis. A composite score based analysis does not use the same implicit weighting of the item-level data, but items that are insensitive to the latent variable (i.e., uninformative) will also not meaningfully contribute when their scores are added together.

In addition to the quantitative arguments considered in this work, other critical aspects such as concordance to full scale, dimensionality, and patient population differences need to be taken into account when reducing scales. In order to determine the utility of shortening an assessment, input from clinicians and disease area experts is essential. Also it is necessary to evaluate a large dataset consisting of multiple trials to more effectively identify which items could be considered for removal in future analysis [20]. From the perspective of drug development it is also important to have trials with positive results to assure no loss in detecting known drug effect when specific items are removed. Noting that in some therapeutic areas for example neurological diseases (i.e. schizophrenia, Alzheimer’s); drug effect may be small and only a few positive trials may exist. The full clinical endpoint scale has been held as the gold standard by which marketed drugs have been approved therefore utilizing shortened assessments for new therapeutics as a primary or secondary endpoint would require much discussion and buy in from regulators. Lastly the shortened assessment should not be viewed as a replacement for the original scale, and the clinical implications of identifying a positive result with the shortened assessment and negative result from the full clinical scale should be well understood. It is important to take into account the IR concepts described above along with these additional considerations to understand the impact of removal of items when interpreting the results.

Our findings could serve as a message of caution against a too simplistic approach when considering shortening an assessment. On the other hand, one could also imagine exploiting the multidimensionality issues described above to increase the sensitivity of the assessment with respect to a particular symptom dimension. If a novel treatment is expected to affect mostly the symptom dimensions measured by “Finger Tap-left hand” and “Hand Move-left hand” then only including items that align well with these items could increase the power of the analysis. A deeper exploration of circumstances when dropping items could be beneficial is a possible extension of this work.

## Conclusion

The IR model was able to successfully estimate parameters using the reduced information and item scenarios. However, interpreting the results and comparing them to the results obtained for the full assessment is more challenging and requires careful consideration of the phenomena described in this work. An understanding of the trade-off between information gained through re-alignment of the average disease disability dimension and information loss due to removal of items is required.

## Electronic supplementary material

Below is the link to the electronic supplementary material.

Supplementary file1 (PDF 519 kb)

Supplementary file2 (PDF 1188 kb)

Supplementary file3 (DOCX 401 kb)
