# Supplementary material for: Performance of longitudinal item response theory models in shortened or partial assessments
Source: J Pharmacokinet Pharmacodyn. 2020 Jul 2;47(5):461–71. doi: 10.1007/s10928-020-09697-x (PMC7520414; doi:10.1007/s10928-020-09697-x)
Supplement: Supplementary file 1 — Supplementary file1 (PDF 519 kb) [file 10928_2020_9697_MOESM1_ESM.pdf]

Supplementary Materials 1

Journal of Pharmacokinetics and Pharmacodynamics

Title: Performance of Longitudinal Item Response Theory Models in Shortened Assessments

Authors: Leticia Arrington[1,2], Sebastian Ueckert[1], Malidi Ahamadi[2], Sreeraj Macha[2] and Mats O. Karlsson[1,3]

[1] Department of Pharmaceutical Biosciences, Uppsala University, P.O. Box 591, 751 24 Uppsala, Sweden
[2]Merck & Co. Inc. Kenilworth, NJ, USA
[3]Corresponding author: email Mats.Karlsson@farmbio.uu.se and phone +46 184 714 105

Table S1 Estimated Population Disease Progression Slope Parameter Values and Uncertainty from the IR-Longitudinal Model for Observed Motor Subscale Data at each level of Information Content (removal of most informative items first)

% Information	Disease Progression Rate (latent variable/yr.)	RSE%[a]	ω[b]	RSE%[a]
100%	0.21	10.8	0.14	13.2
80%	0.22	10.9	0.15	13.4
70%	0.22	11.1	0.15	13.9
60%	0.21	12.0	0.15	15.6
50%	0.23	10.0	0.13	14.5
40%	0.22	11.0	0.13	15.7
30%	0.21	11.0	0.11	17.3
20%	0.20	11.3	0.09	19.9
10%	0.18	12.9	0.06	24.9

[a]Relative Standard Error is the SE/ parameter estimate *100
[b]Variance of Random Effect

Table S2 Estimated Population Disease Progression Slope Parameter Values and Uncertainty from the IR-Longitudinal Model for Observed Motor Subscale Data at each level of Information Content (removal of least informative items first)

% Information	Disease Progression Rate (latent variable/yr.)	RSE%[a]	ω[b]	RSE%[a]
100%	0.21	10.8	0.14	13.2
90%	0.20	11.4	0.14	13.7
80%	0.20	11.1	0.13	14.2
70%	0.19	11.3	0.12	14.8
60%	0.19	10.7	0.10	15.1
50%	0.17	11.7	0.10	15.6
40%	0.19	10.1	0.08	15.7
30%	0.17	11.5	0.08	17.8
20%	0.16	12.6	0.07	19.5

[a]Relative Standard Error is the SE/ parameter estimate *100
[b]Variance of Random Effect

Table S3 Estimated Population Disease Progression Slope and Drug Effect Parameter Values and Uncertainty from the IR-Longitudinal Model for Observed Motor Subscale Data at each level of Information Content (removal of most informative items first)

% Information	Disease Progression Rate (latent variable/yr.) (RSE%[a])	ω[b](RSE%)	Symptomatic Drug Effect (RSE%[a])	ω[b](RSE%)
100%	0.38 (7.6)	0.12 (15.8)	-0.39 (12.2)	0.39 (16.6)
80%	0.4 (7.2)	0.12 (15.2)	-0.41 (11.8)	0.39 (15.6)
70%	0.4 (7.5)	0.12 (16.2)	-0.42 (11.9)	0.36 (15.9)
60%	0.41 (7.3)	0.12 (16.1)	-0.45 (11.4)	0.36 (15.2)
50%	0.4 (7.5)	0.1 (17.1)	-0.39 (12.7)	0.32 (16.7)
40%	0.4 (7.8)	0.1 (18)	-0.42 (12.5)	0.3 (16.9)
30%	0.36 (8.6)	0.08 (21.2)	-0.38 (14)	0.27 (19.6)
20%	0.34 (9.1)	0.06 (23.7)	-0.35 (15.3)	0.19 (24.5)
10%	0.27 (10.6)	0.04 (33.7)	-0.24 (20.6)	0.1 (41.3)

[a]Relative Standard Error is the SE/ parameter estimate *100
[b]Variance of Random Effect

Table S4 Estimated Population Disease Progression Slope and Drug Effect Parameter Values and Uncertainty from the IR-Longitudinal Model for Observed Motor Subscale Data at each level of Information Content (removal of least informative items first)

% Information	Disease Progression Rate (latent variable/yr.) (RSE%[a])	ω[b](RSE%)	Symptomatic Drug Effect (RSE%[a])	ω[b](RSE%)
100%	0.38 (7.3)	0.12 (15.3)	-0.39 (12.1)	0.39 (16)
90%	0.36 (7.5)	0.12 (15.4)	-0.38 (12.3)	0.36 (16.5)
80%	0.34 (7.6)	0.1 (15.3)	-0.33 (13.4)	0.33 (16.9)
70%	0.33 (8.1)	0.1 (15.8)	-0.32 (14.2)	0.31 (18.1)
60%	0.31 (8.2)	0.08 (16.5)	-0.3 (15.1)	0.27 (19.1)
50%	0.31 (8.5)	0.08 (17.4)	-0.32 (14.4)	0.26 (18.8)

**40%**	0.28 (8.9)	0.06 (17.8)	-0.23 (18.9)	0.2 (19.9)
**30%**	0.26 (10.2)	0.06 (20)	-0.23 (19.7)	0.19 (21.3)
**20%**	0.24 (11.3)	0.05 (23.3)	-0.22 (21.8)	0.15 (25.5)

[a]**Relative Standard Error is the SE/ parameter estimate *100**
[b]**Variance of Random Effect**
