# Supplementary material for: Performance of longitudinal item response theory models in shortened or partial assessments
Source: J Pharmacokinet Pharmacodyn. 2020 Jul 2;47(5):461–71. doi: 10.1007/s10928-020-09697-x (PMC7520414; doi:10.1007/s10928-020-09697-x)
Supplement: Supplementary file 2 — Supplementary file2 (PDF 1188 kb) [file 10928_2020_9697_MOESM2_ESM.pdf]

Supplementary Materials 2

Journal of Pharmacokinetics and Pharmacodynamics
Title: Performance of Longitudinal Item Response Theory Models in Shortened Assessments
Authors: Leticia Arrington[1,2,] Sebastian Ueckert[1], Malidi Ahamadi[2*], Sreeraj Macha[2*] and Mats O. Karlsson[1,3]
1 Department of Pharmaceutical Biosciences, Uppsala University, P.O. Box 591, 751 24 Uppsala, Sweden
2Merck & Co. Inc. Kenilworth, NJ, USA
3Corresponding author: email Mats.Karlsson@farmbio.uu.se and phone +46 184 714 105
*Affiliation at time of work

This appendix contains diagnostic plots (i.e. Categorical VPC and ICC) evaluating the performance of the 100% information scenario from a unidimensional IRT model using simultaneous approach.

Figure S1. Categorical Visual predictive checks (VPCs) describing the time-courses of each score for the MDS- UPDR motor item. Median (blue solid line) of the observed data is compared to the 95% prediction interval (gray shaded area) for the simulated data

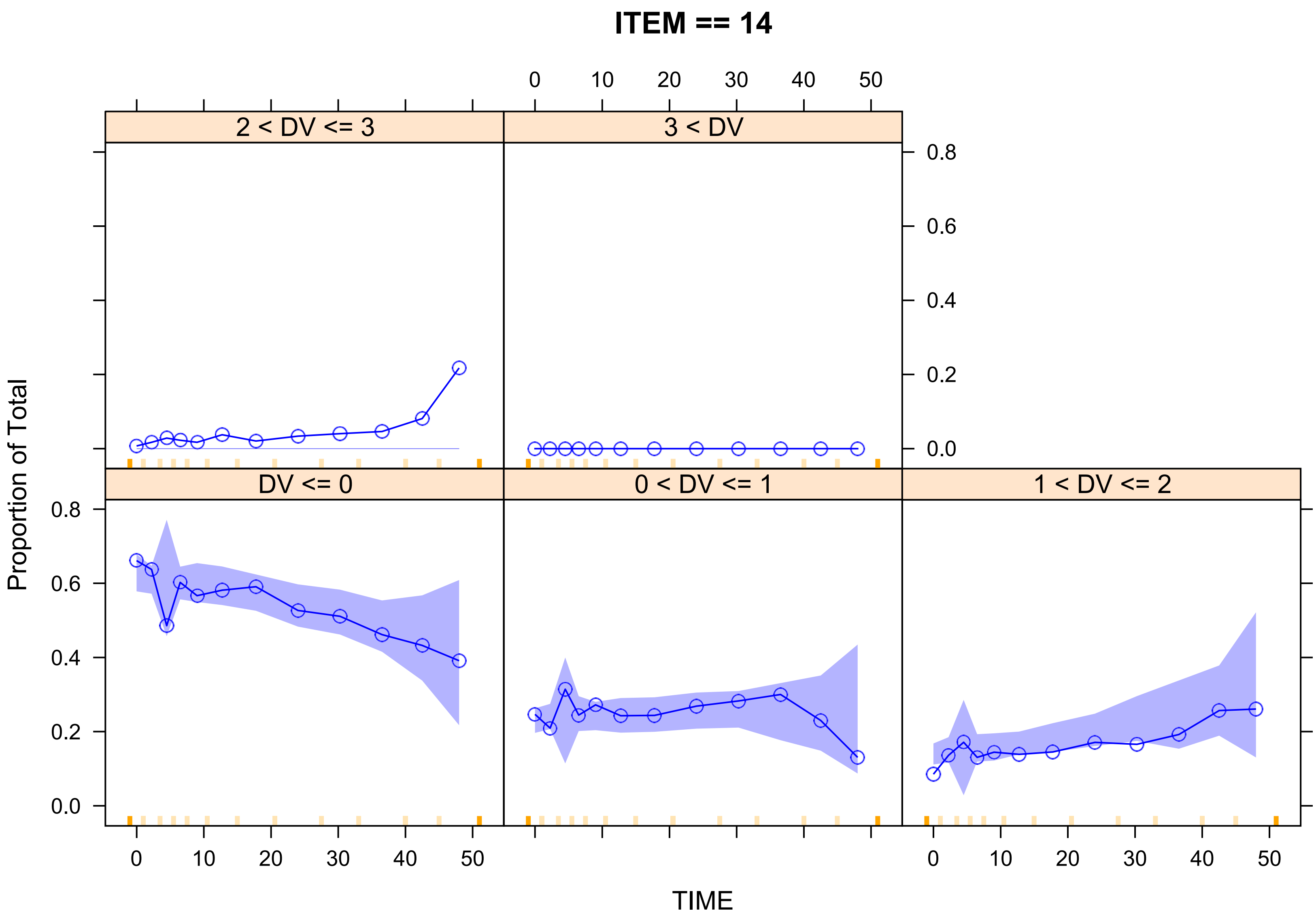

ITEM == 14
2 < DV <= 3
3 < DV
DV <= 0
0 < DV <= 1
1 < DV <= 2
Proportion of Total
TIME
0.0
0.2
0.4
0.6
0.8
0
10
20
30
40
50

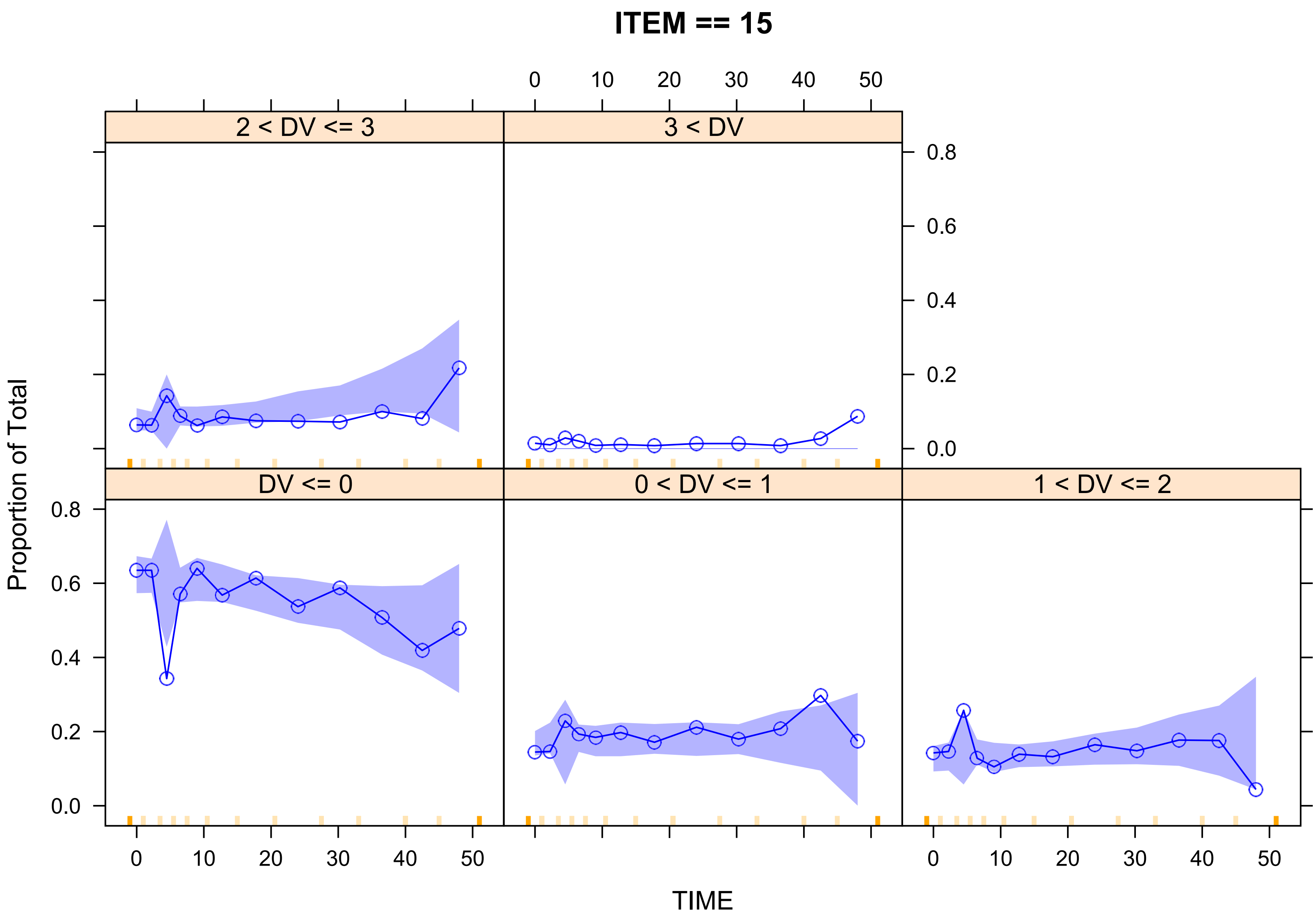
ITEM == 15
2 < DV <= 3
3 < DV
DV <= 0
0 < DV <= 1
1 < DV <= 2
Proportion of Total
TIME
0.0
0.2
0.4
0.6
0.8
0
10
20
30
40
50

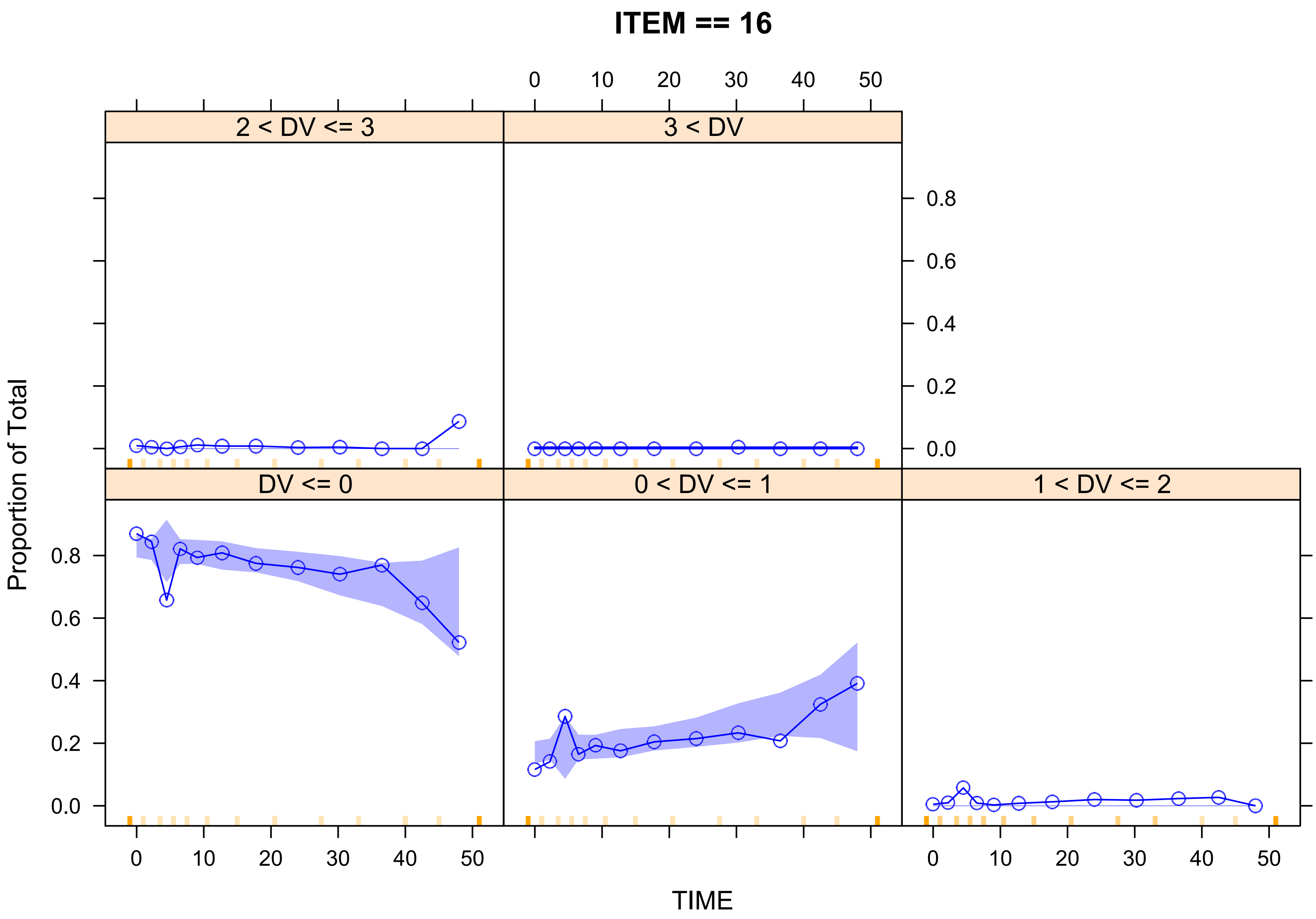

ITEM == 16
2 < DV <= 3
3 < DV
DV <= 0
0 < DV <= 1
1 < DV <= 2
Proportion of Total
TIME
0.0
0.2
0.4
0.6
0.8
0
10
20
30
40
50

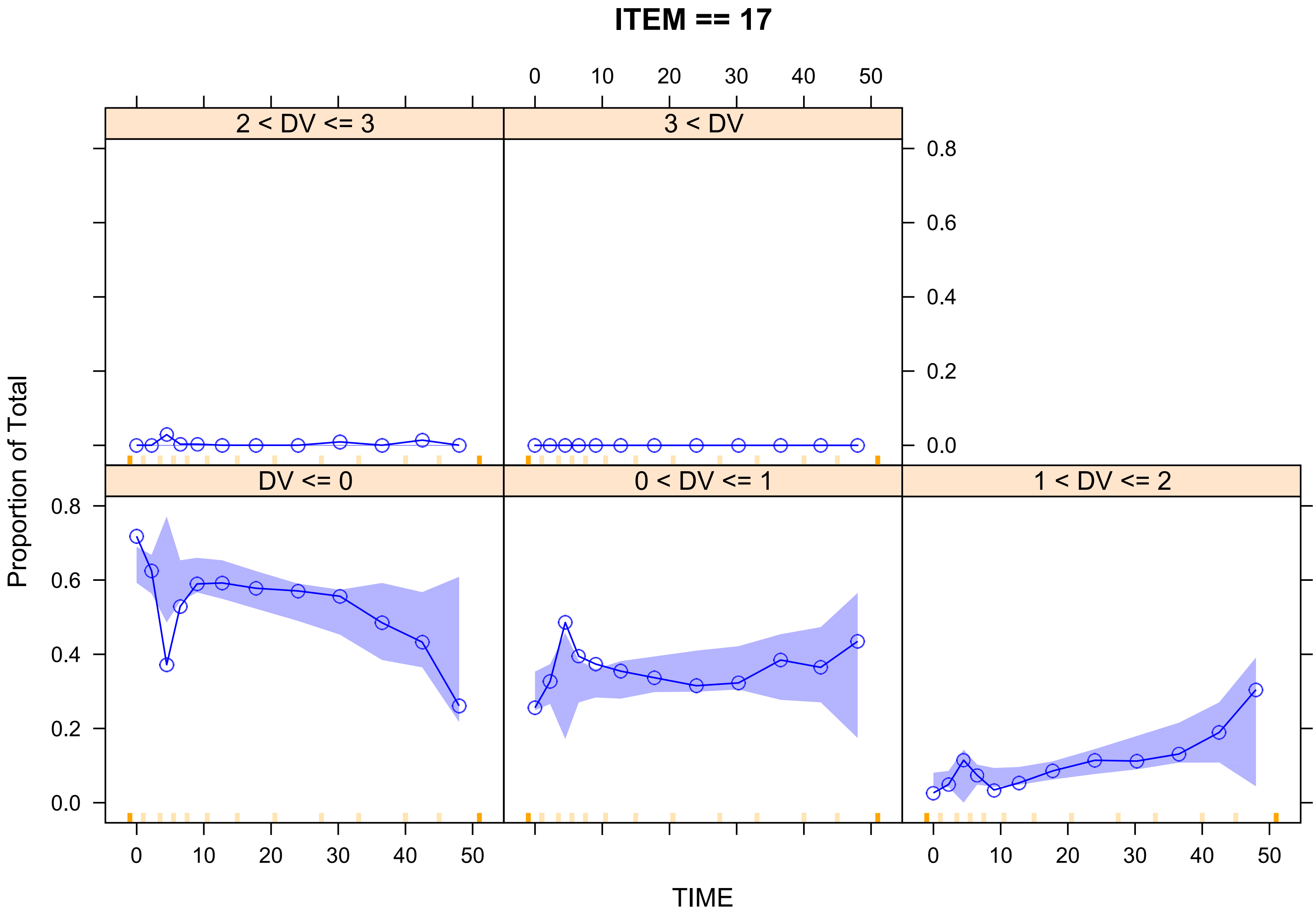
ITEM == 17
2 < DV <= 3
3 < DV
DV <= 0
0 < DV <= 1
1 < DV <= 2
Proportion of Total
TIME
0.0
0.2
0.4
0.6
0.8
0
10
20
30
40
50

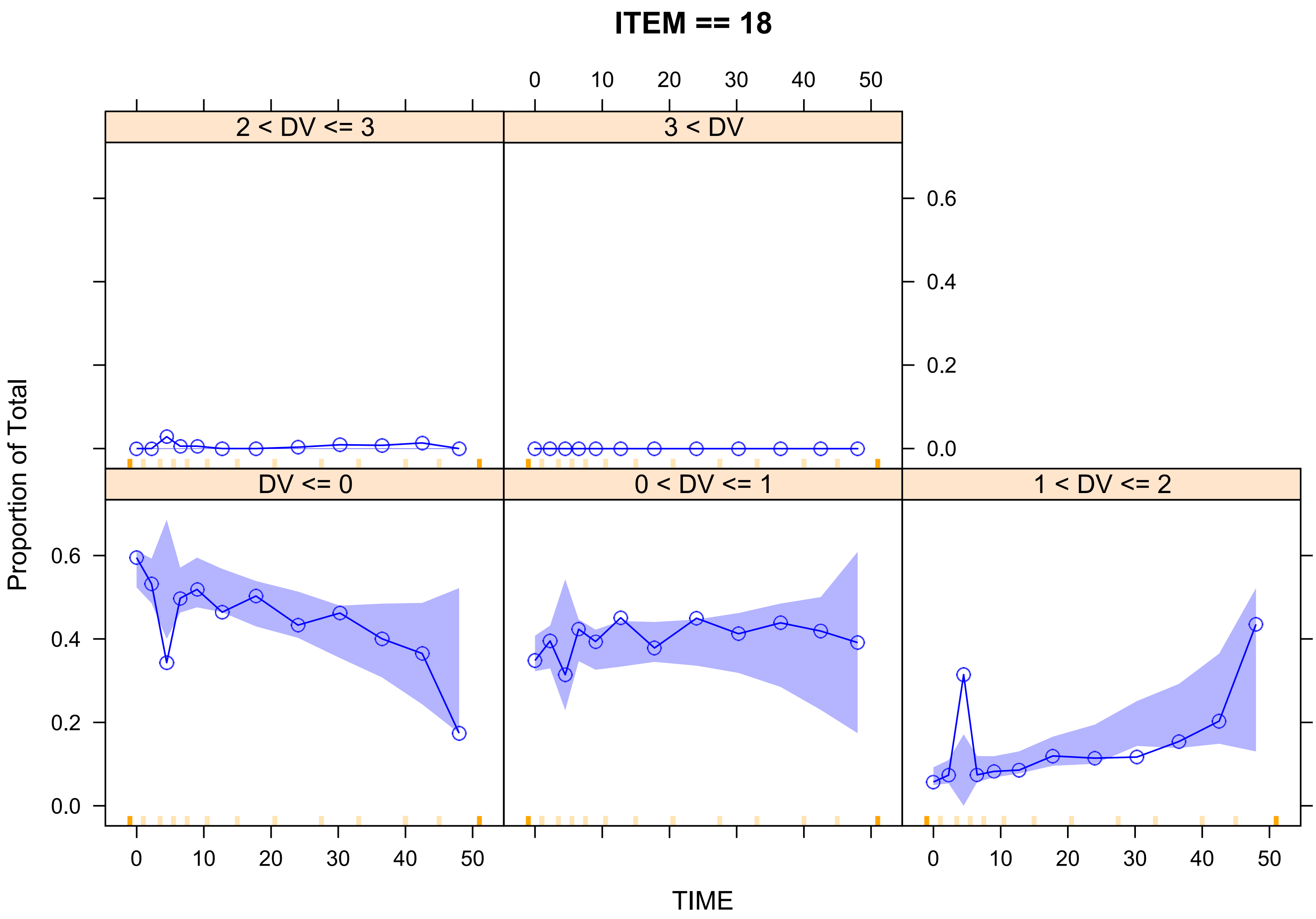

ITEM == 18
2 < DV <= 3
3 < DV
DV <= 0
0 < DV <= 1
1 < DV <= 2
Proportion of Total
TIME
0.0
0.2
0.4
0.6
0
10
20
30
40
50

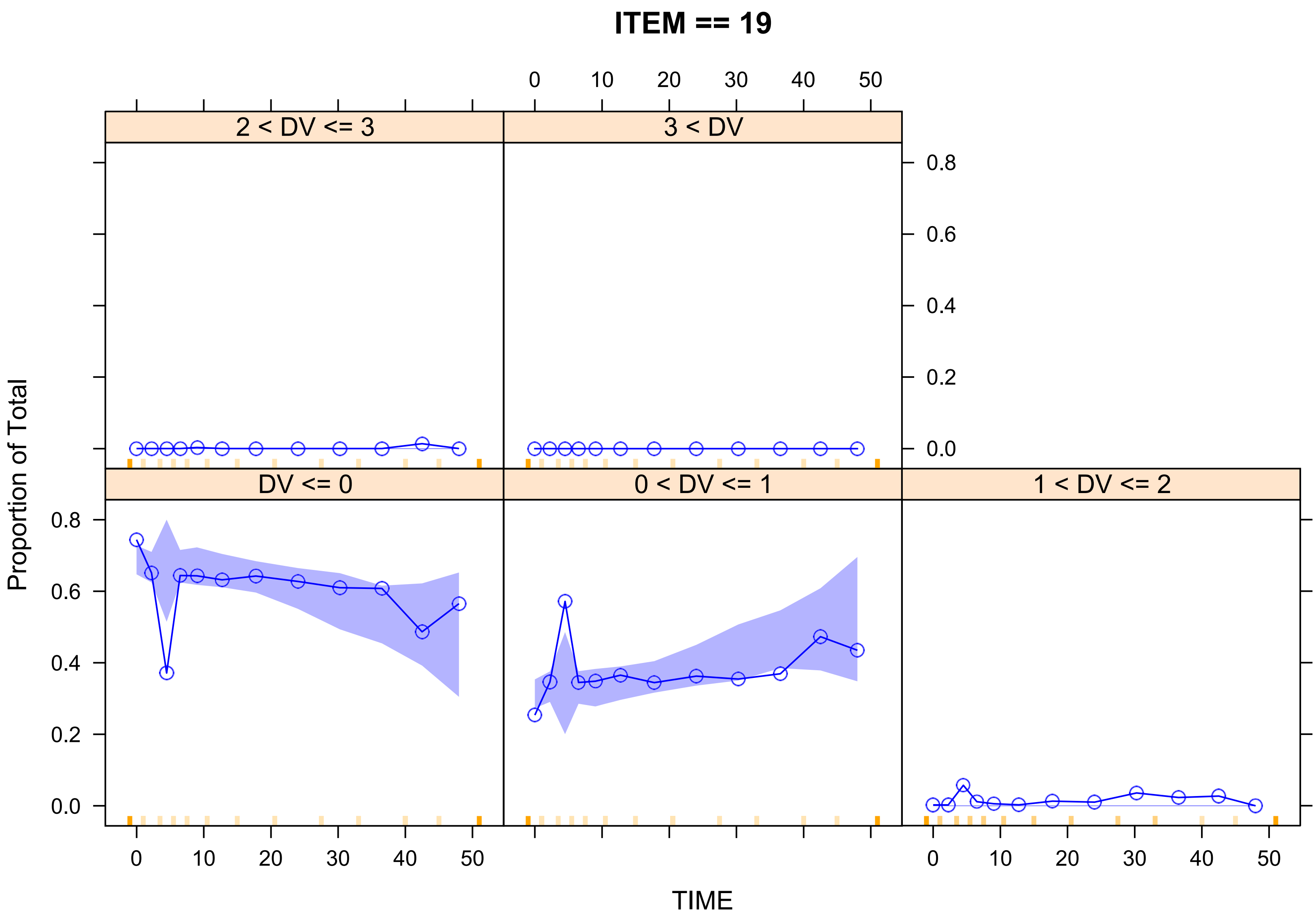
ITEM == 19
2 < DV <= 3
3 < DV
DV <= 0
0 < DV <= 1
1 < DV <= 2
Proportion of Total
TIME
0.0
0.2
0.4
0.6
0.8
0
10
20
30
40
50

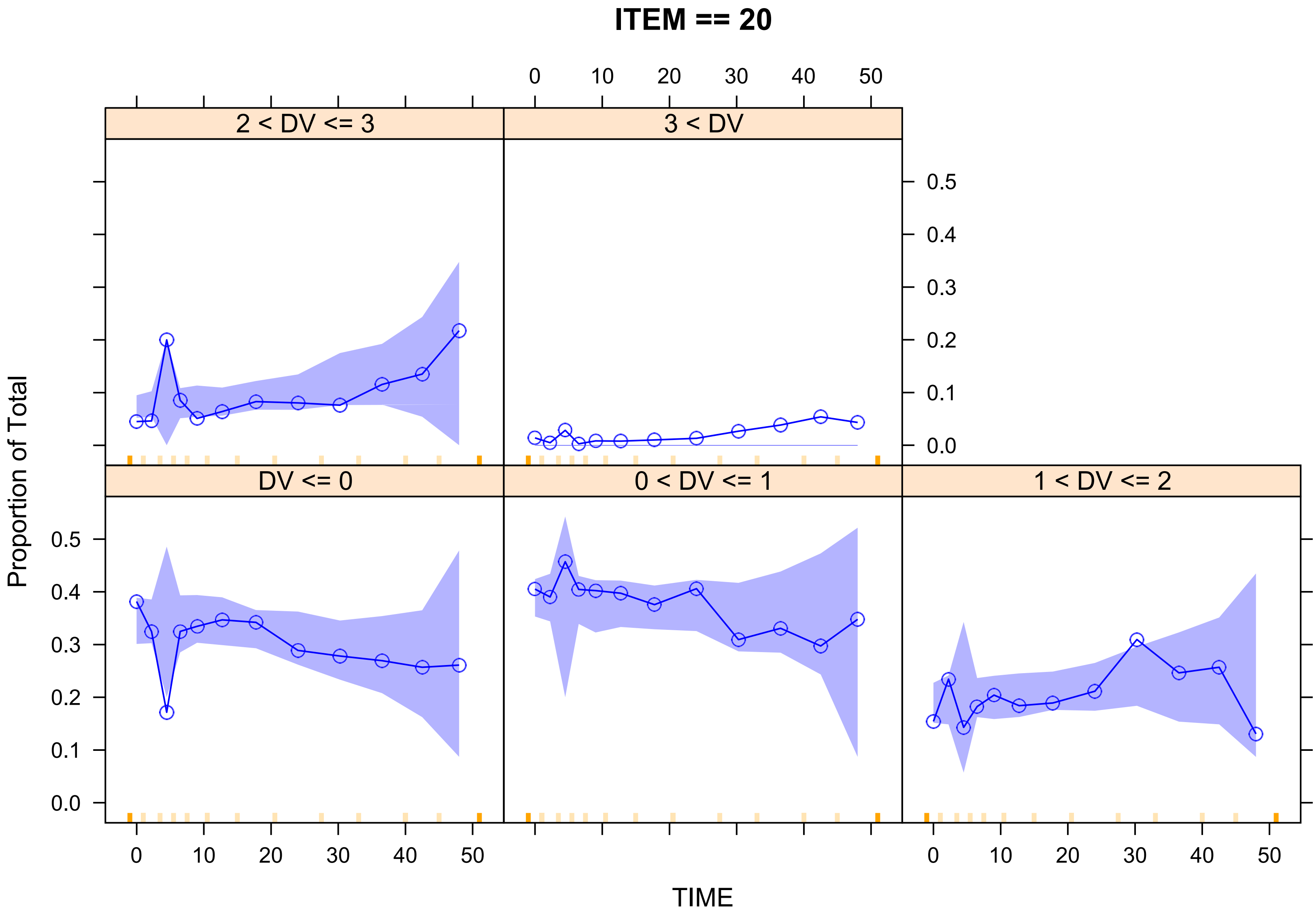

ITEM == 20
2 < DV <= 3
3 < DV
DV <= 0
0 < DV <= 1
1 < DV <= 2
Proportion of Total
TIME
0.0
0.1
0.2
0.3
0.4
0.5
0
10
20
30
40
50

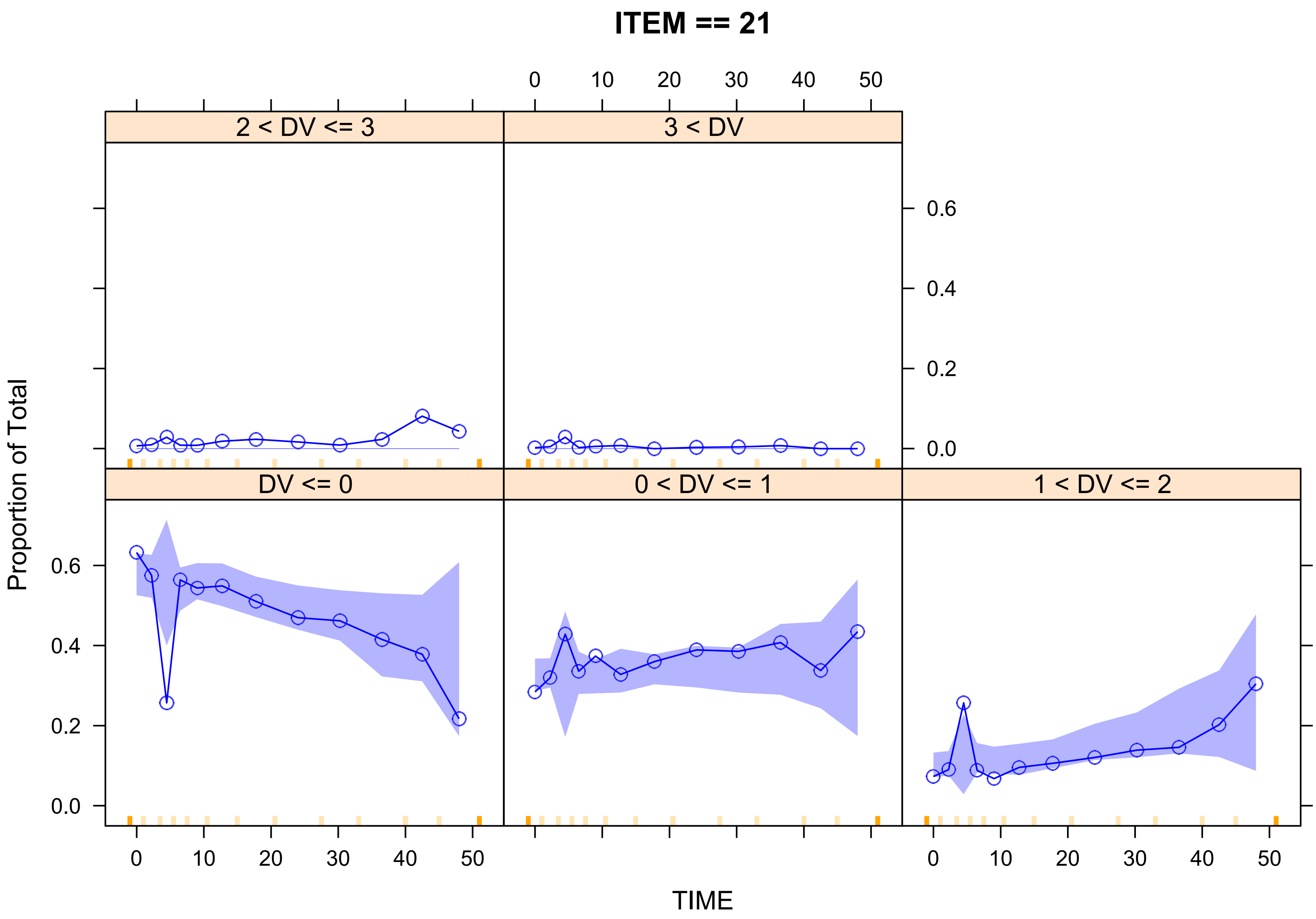

ITEM == 21
2 < DV <= 3
3 < DV
DV <= 0
0 < DV <= 1
1 < DV <= 2
Proportion of Total
TIME
0.0
0.2
0.4
0.6
0
10
20
30
40
50

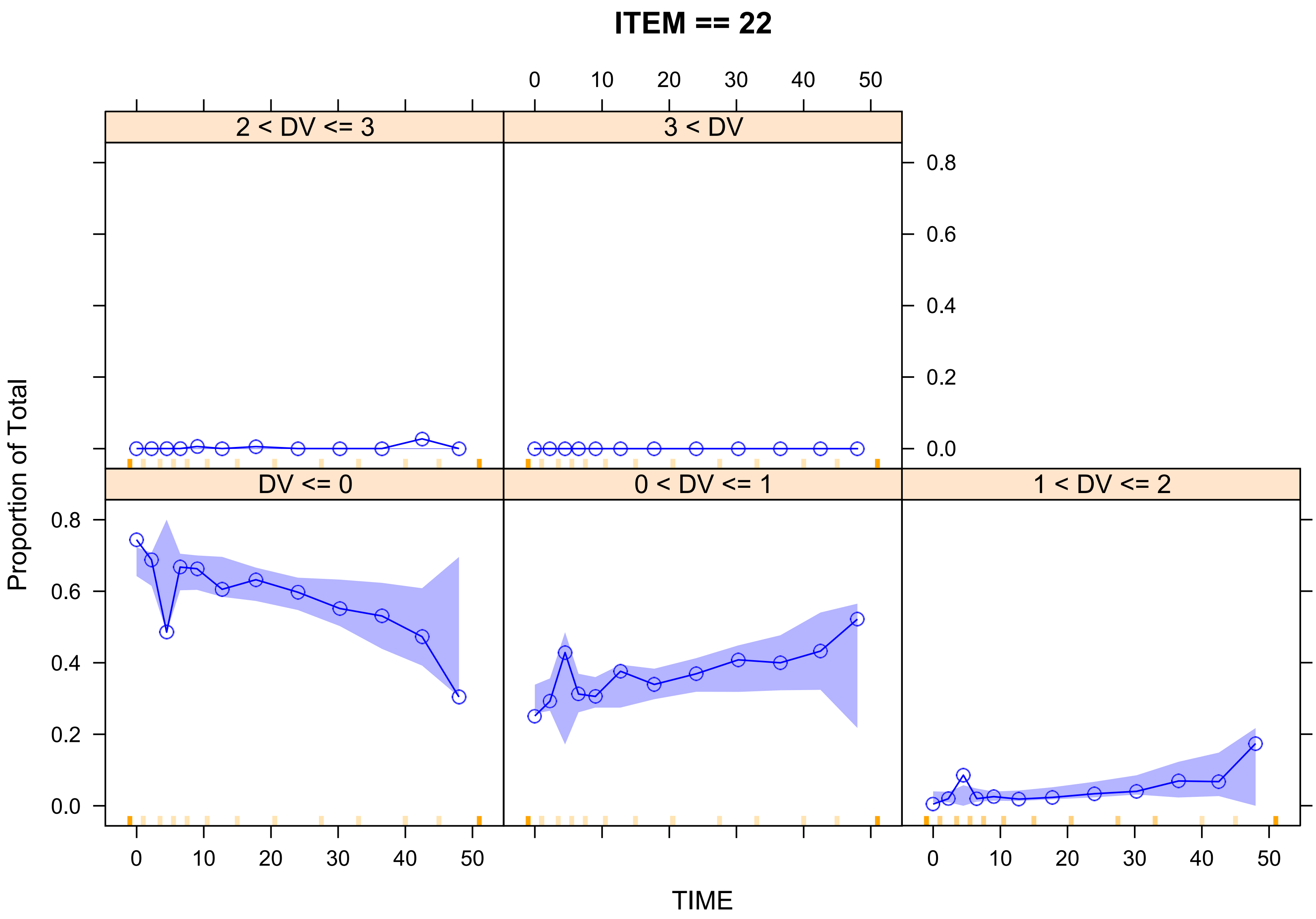

ITEM == 22
2 < DV <= 3
3 < DV
DV <= 0
0 < DV <= 1
1 < DV <= 2
Proportion of Total
TIME
0.0
0.2
0.4
0.6
0.8
0
10
20
30
40
50

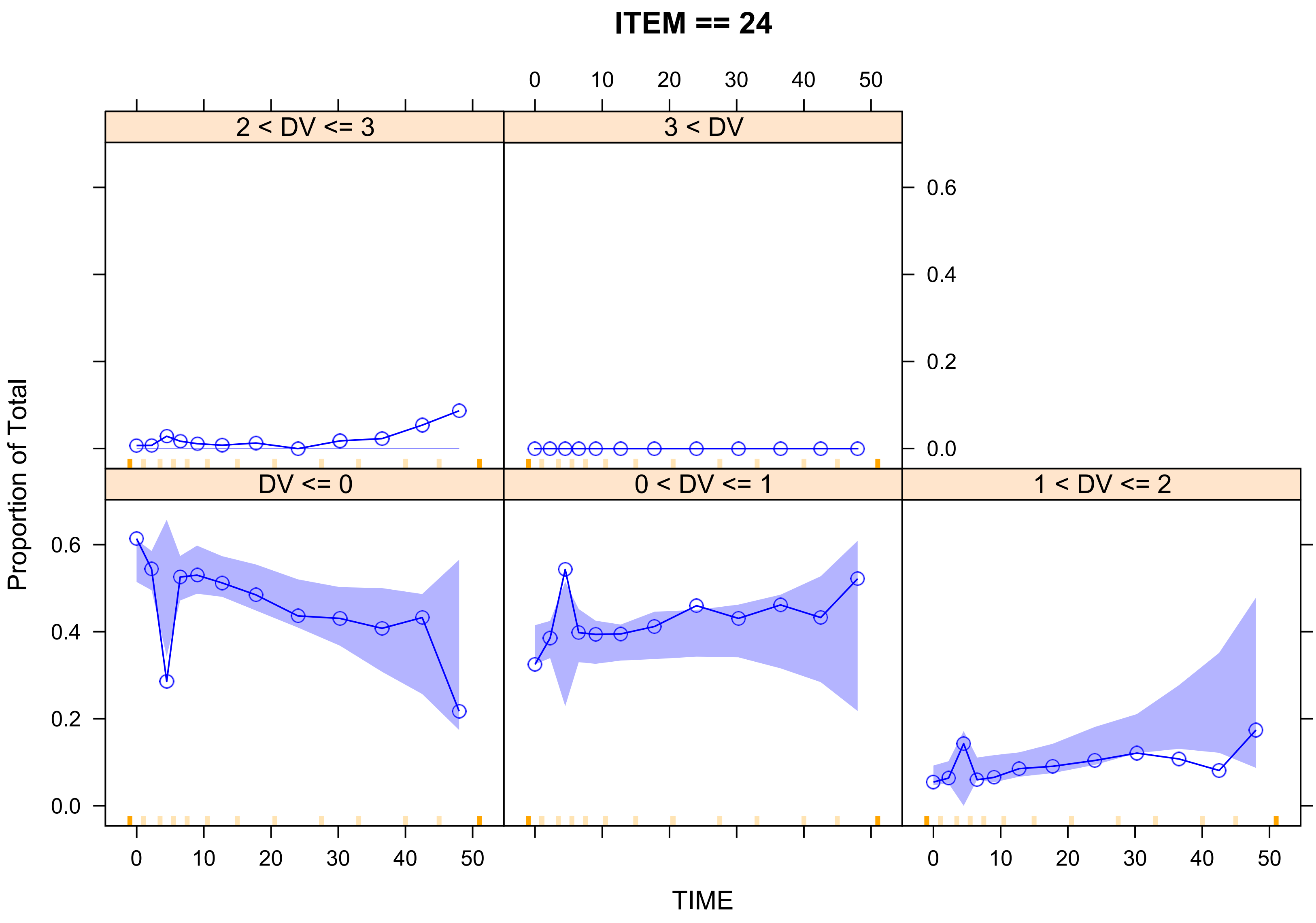
ITEM == 24
2 < DV <= 3
3 < DV
DV <= 0
0 < DV <= 1
1 < DV <= 2
Proportion of Total
TIME
0.0
0.2
0.4
0.6
0
10
20
30
40
50

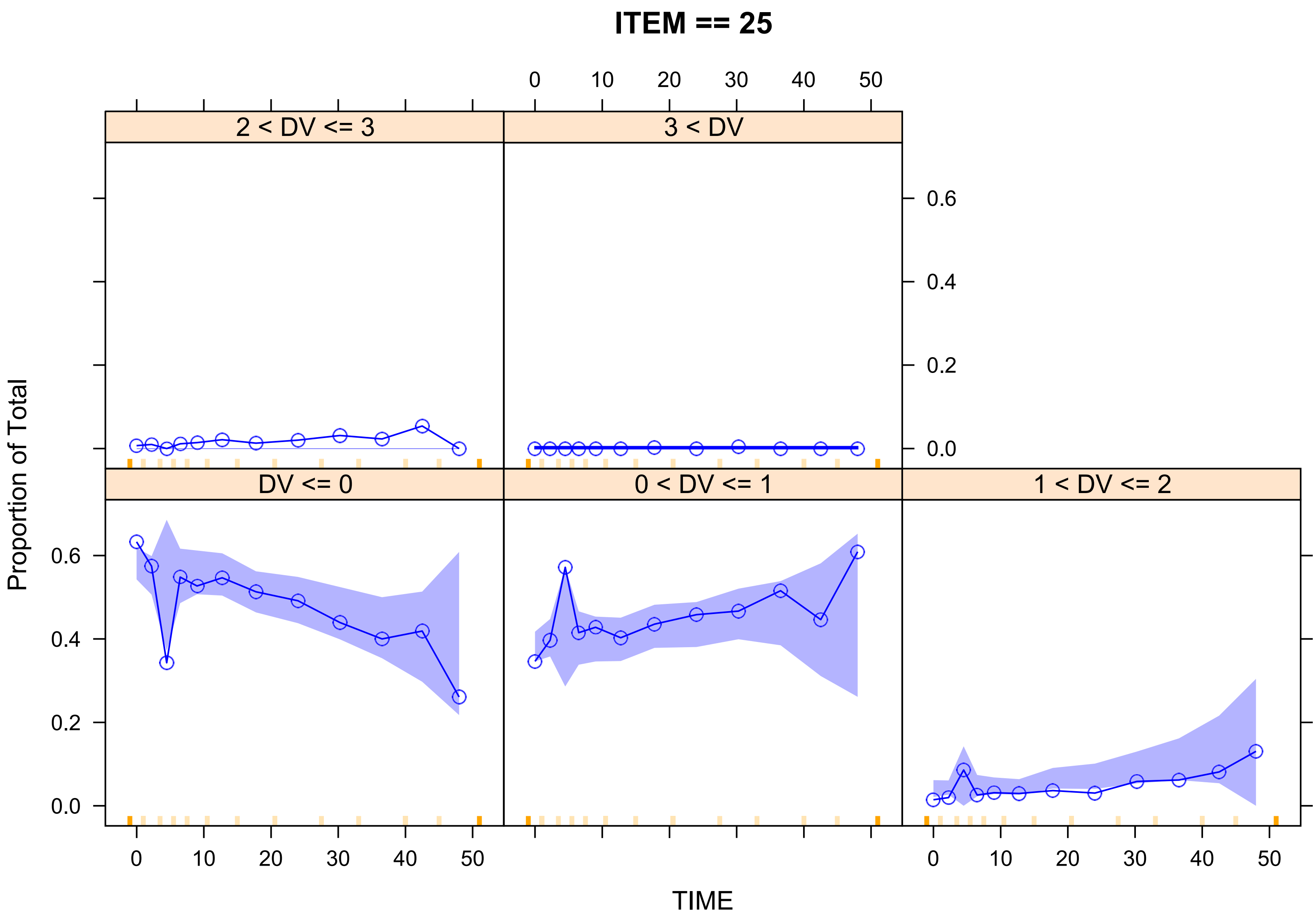

ITEM == 25
2 < DV <= 3
3 < DV
DV <= 0
0 < DV <= 1
1 < DV <= 2
Proportion of Total
TIME
0
10
20
30
40
50
0.0
0.2
0.4
0.6

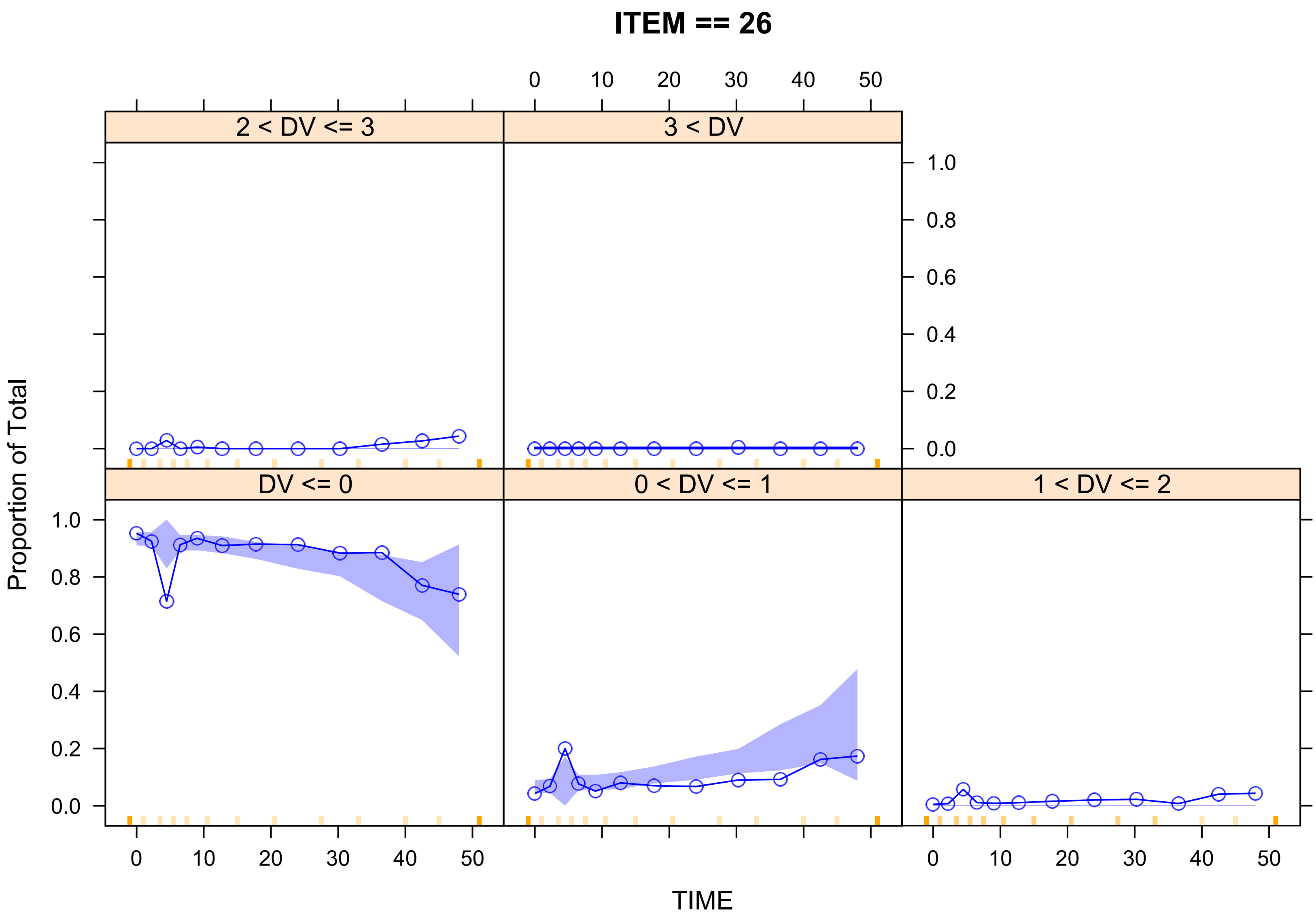
ITEM == 26
2 < DV <= 3
3 < DV
DV <= 0
0 < DV <= 1
1 < DV <= 2
Proportion of Total
TIME
0.0
0.2
0.4
0.6
0.8
1.0
0
10
20
30
40
50

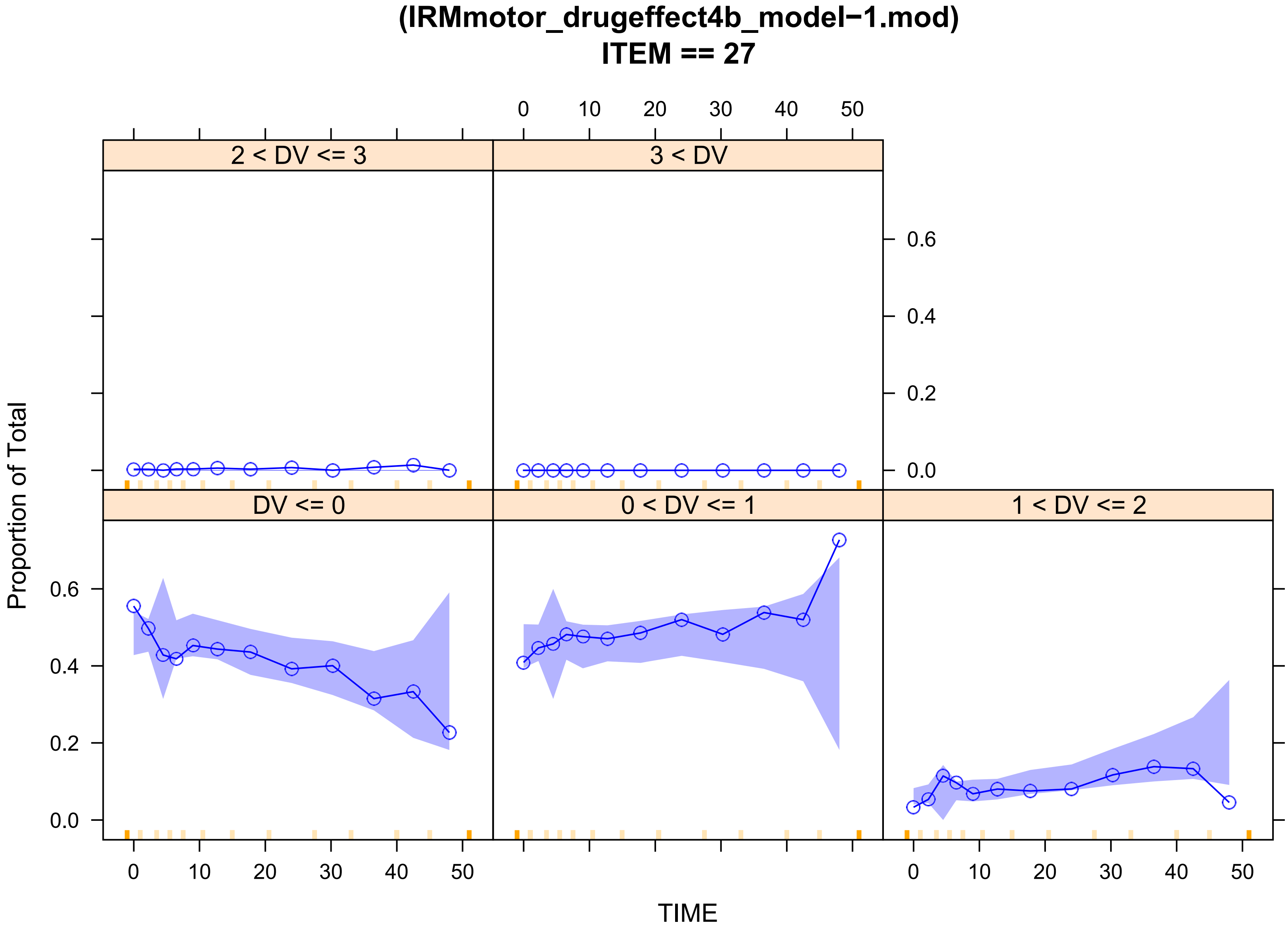

(IRMmotor_drugeffect4b_model−1.mod)
ITEM == 27
2 < DV <= 3
3 < DV
DV <= 0
0 < DV <= 1
1 < DV <= 2
Proportion of Total
TIME
0.0
0.2
0.4
0.6
0
10
20
30
40
50

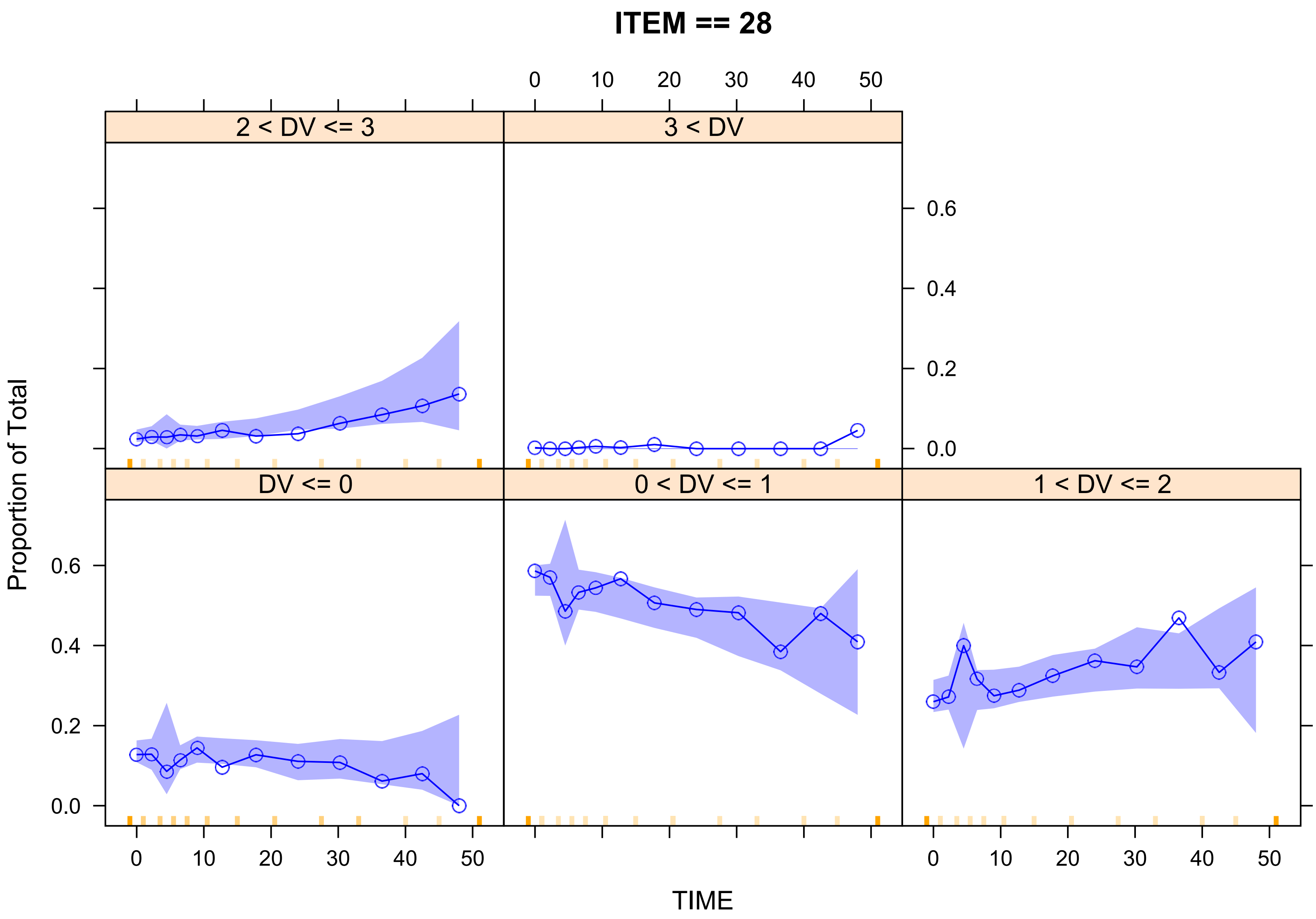

ITEM == 28
2 < DV <= 3
3 < DV
DV <= 0
0 < DV <= 1
1 < DV <= 2
Proportion of Total
TIME
0.0
0.2
0.4
0.6
0
10
20
30
40
50

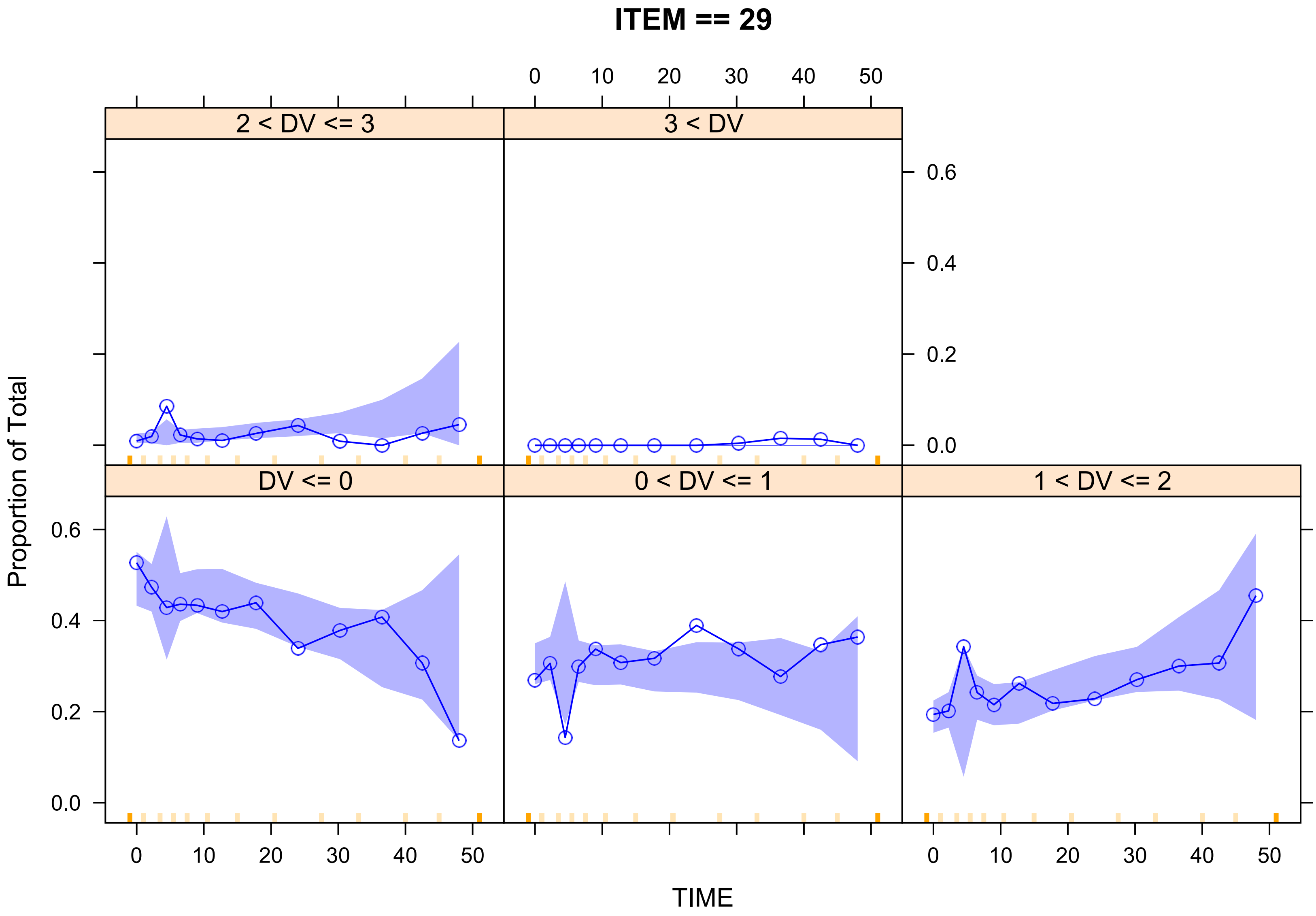
ITEM == 29
2 < DV <= 3
3 < DV
DV <= 0
0 < DV <= 1
1 < DV <= 2
Proportion of Total
TIME
0
10
20
30
40
50
0.0
0.2
0.4
0.6

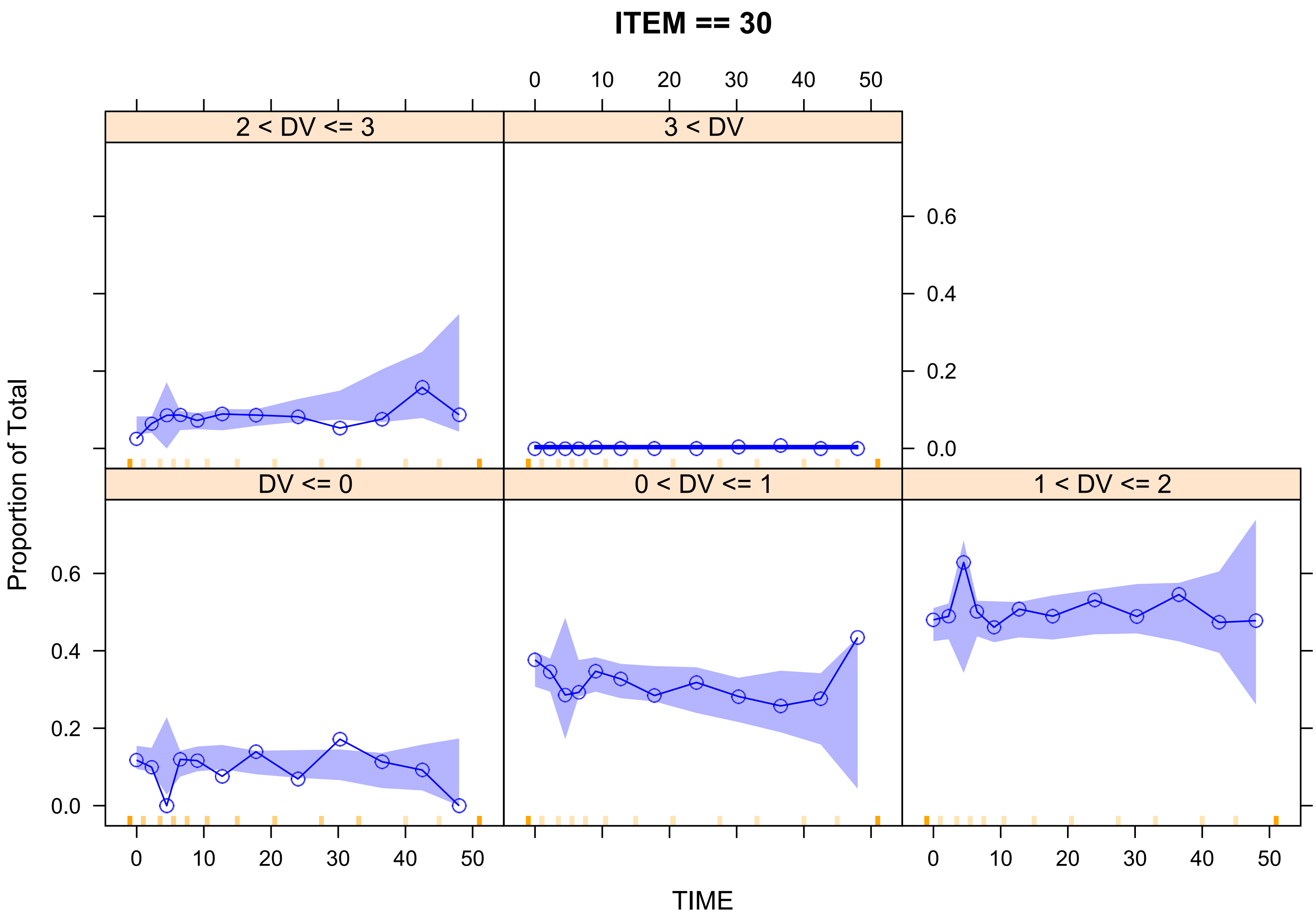
ITEM == 30
2 < DV <= 3
3 < DV
DV <= 0
0 < DV <= 1
1 < DV <= 2
Proportion of Total
TIME
0.0
0.2
0.4
0.6
0
10
20
30
40
50

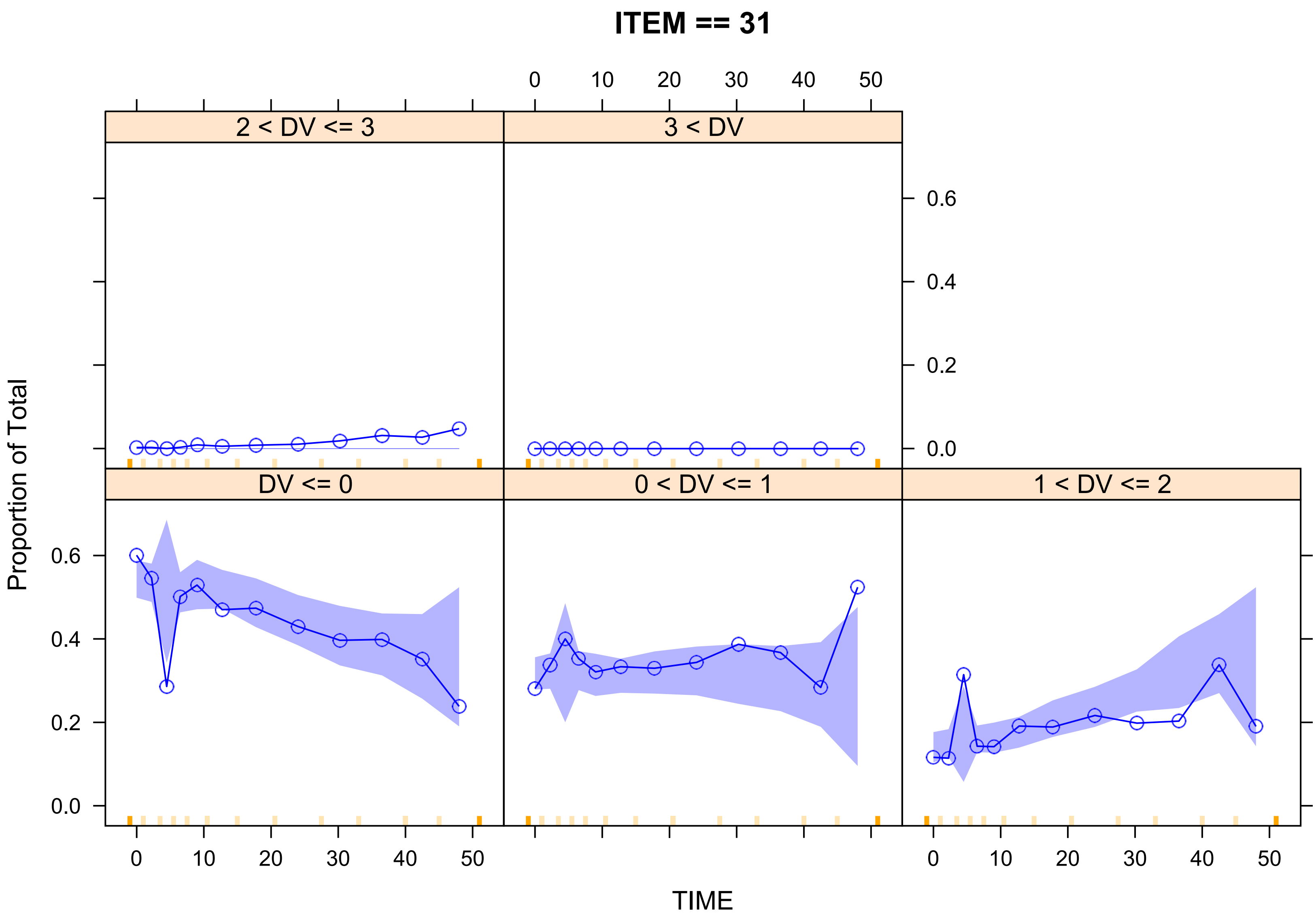
ITEM == 31
2 < DV <= 3
3 < DV
DV <= 0
0 < DV <= 1
1 < DV <= 2
Proportion of Total
TIME
0.0
0.2
0.4
0.6
0
10
20
30
40
50

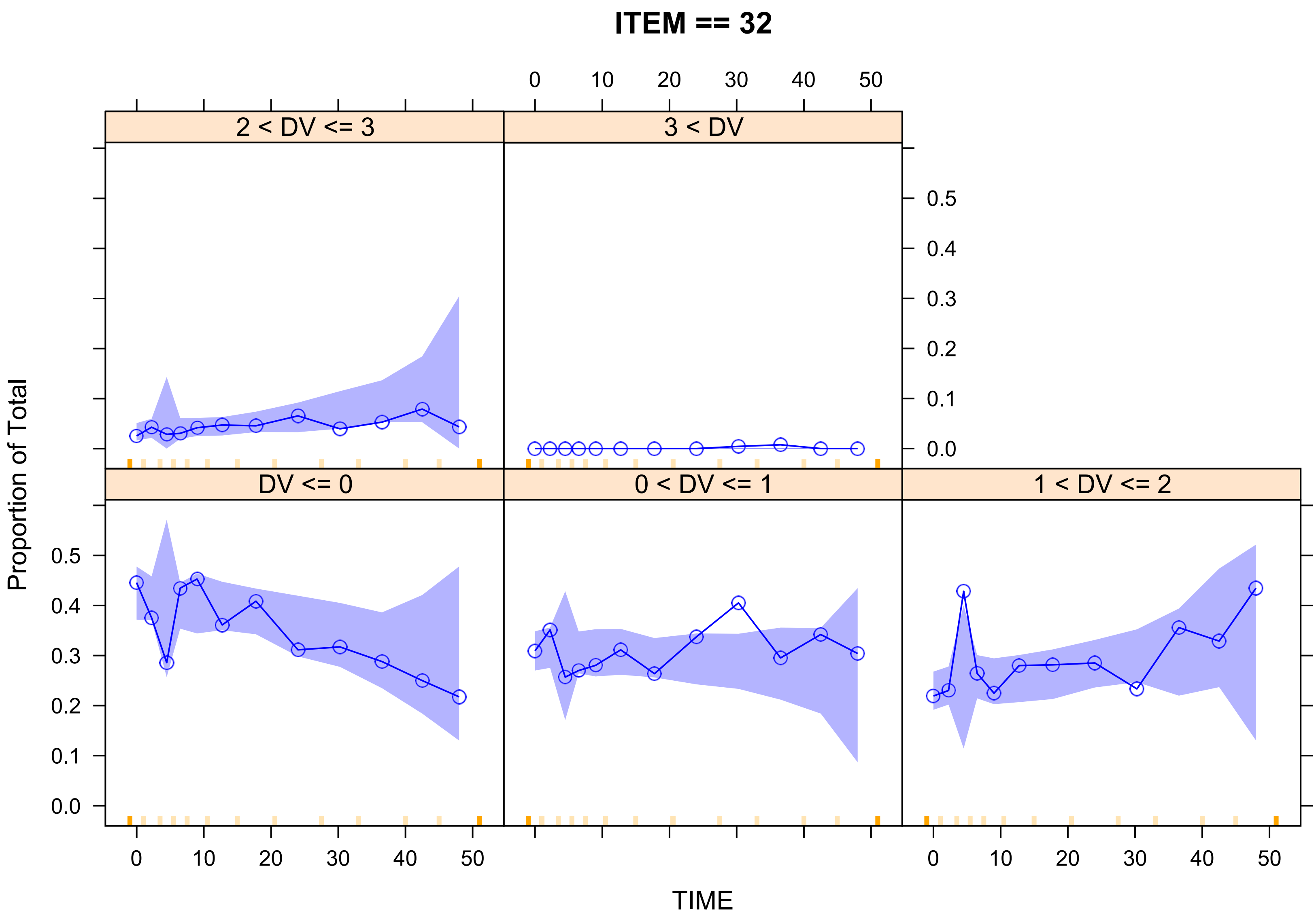
ITEM == 32
2 < DV <= 3
3 < DV
DV <= 0
0 < DV <= 1
1 < DV <= 2
Proportion of Total
TIME
0.0
0.1
0.2
0.3
0.4
0.5
0
10
20
30
40
50

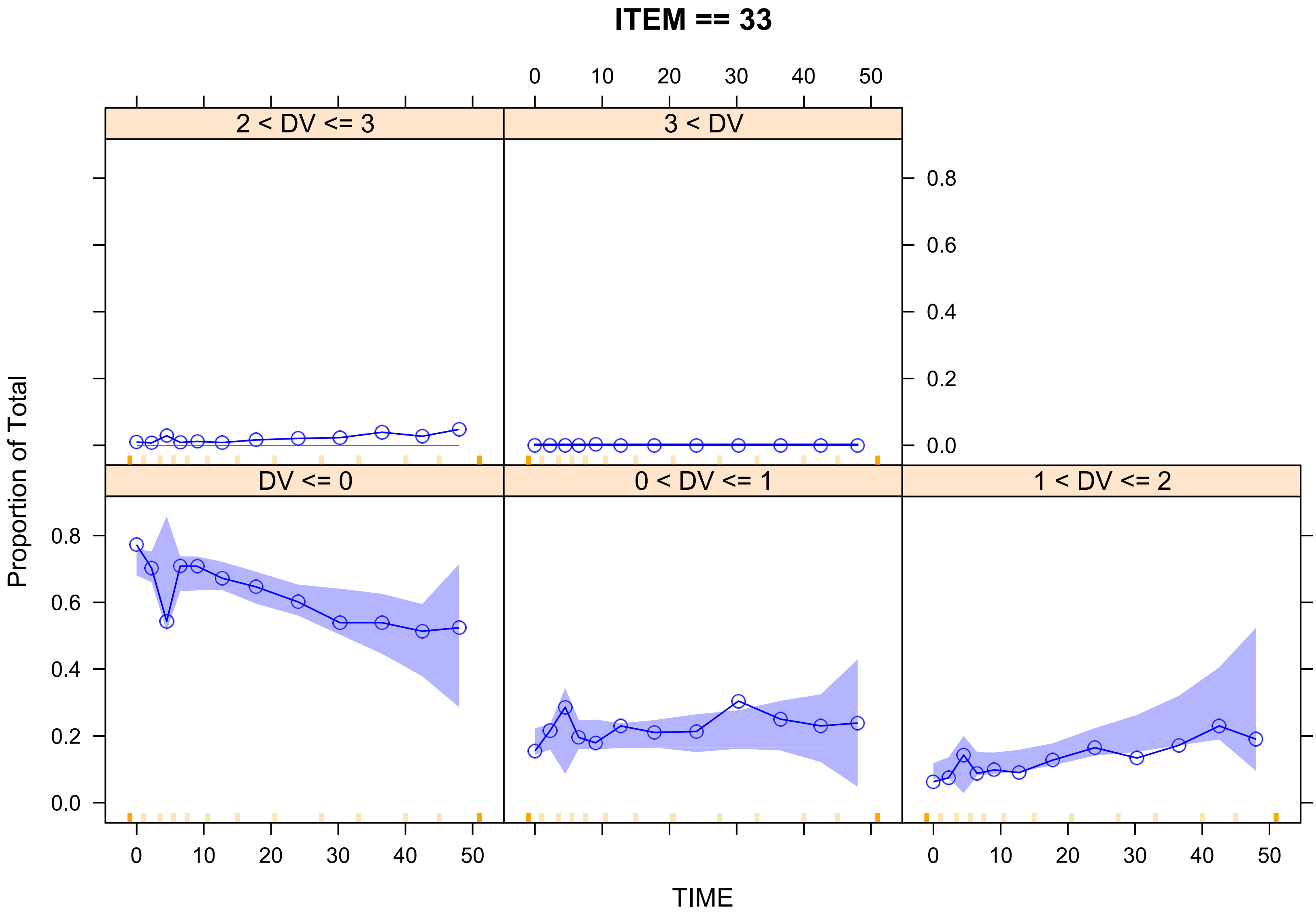

ITEM == 33
2 < DV <= 3
3 < DV
DV <= 0
0 < DV <= 1
1 < DV <= 2
Proportion of Total
TIME
0.0
0.2
0.4
0.6
0.8
0
10
20
30
40
50

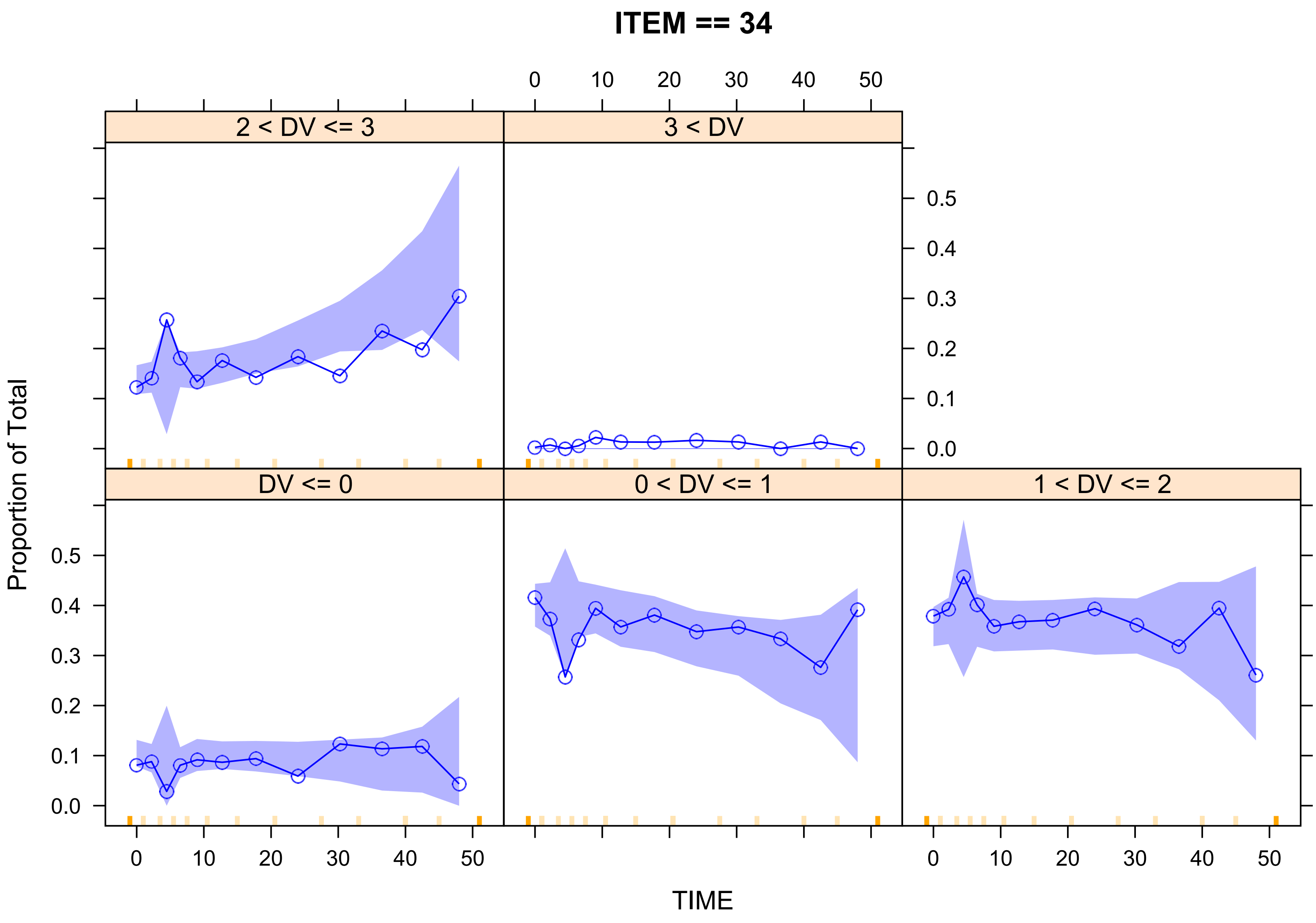

ITEM == 34
2 < DV <= 3
3 < DV
DV <= 0
0 < DV <= 1
1 < DV <= 2
Proportion of Total
TIME
0
10
20
30
40
50
0.0
0.1
0.2
0.3
0.4
0.5

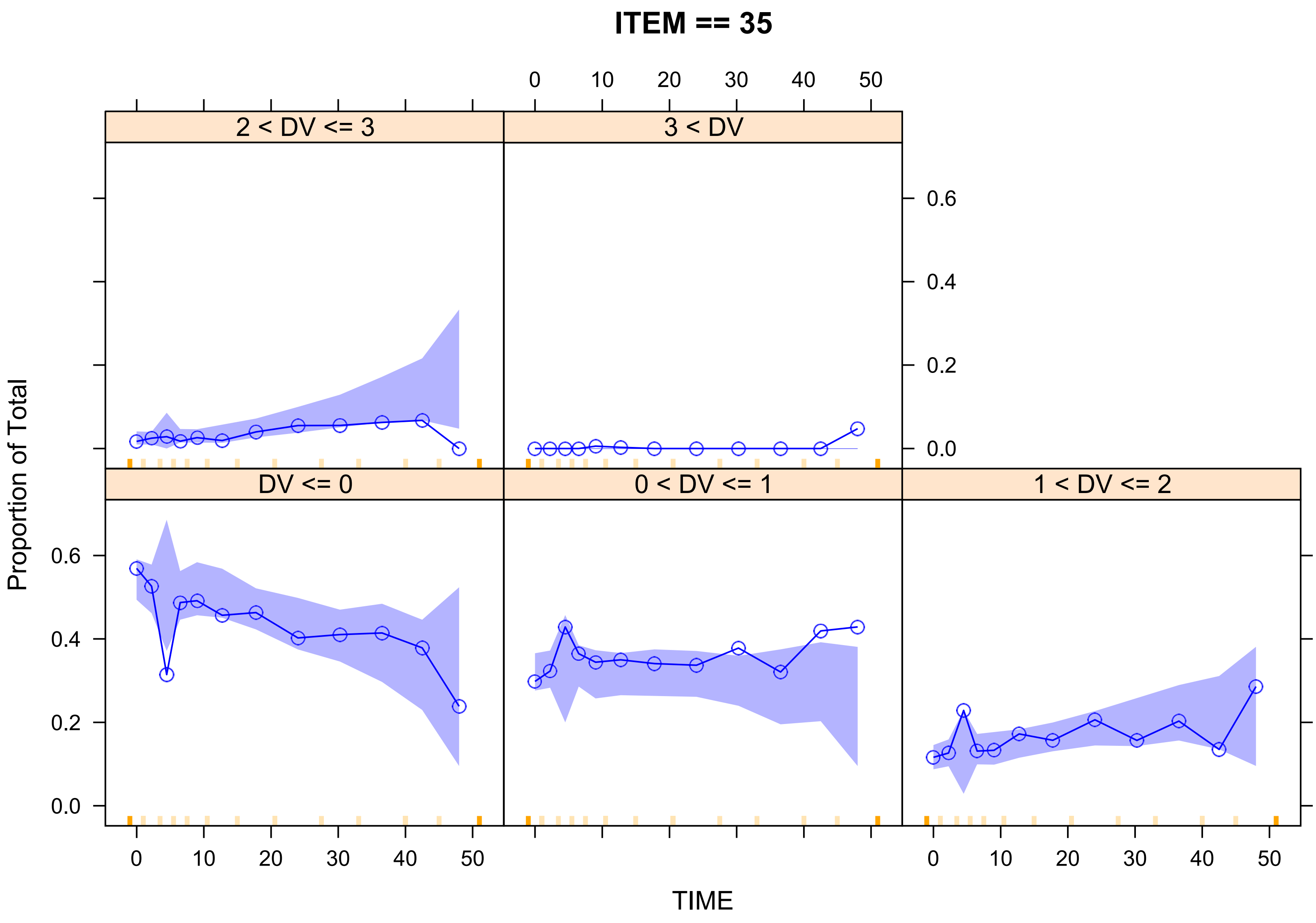

ITEM == 35
2 < DV <= 3
3 < DV
DV <= 0
0 < DV <= 1
1 < DV <= 2
Proportion of Total
TIME
0.0
0.2
0.4
0.6
0
10
20
30
40
50

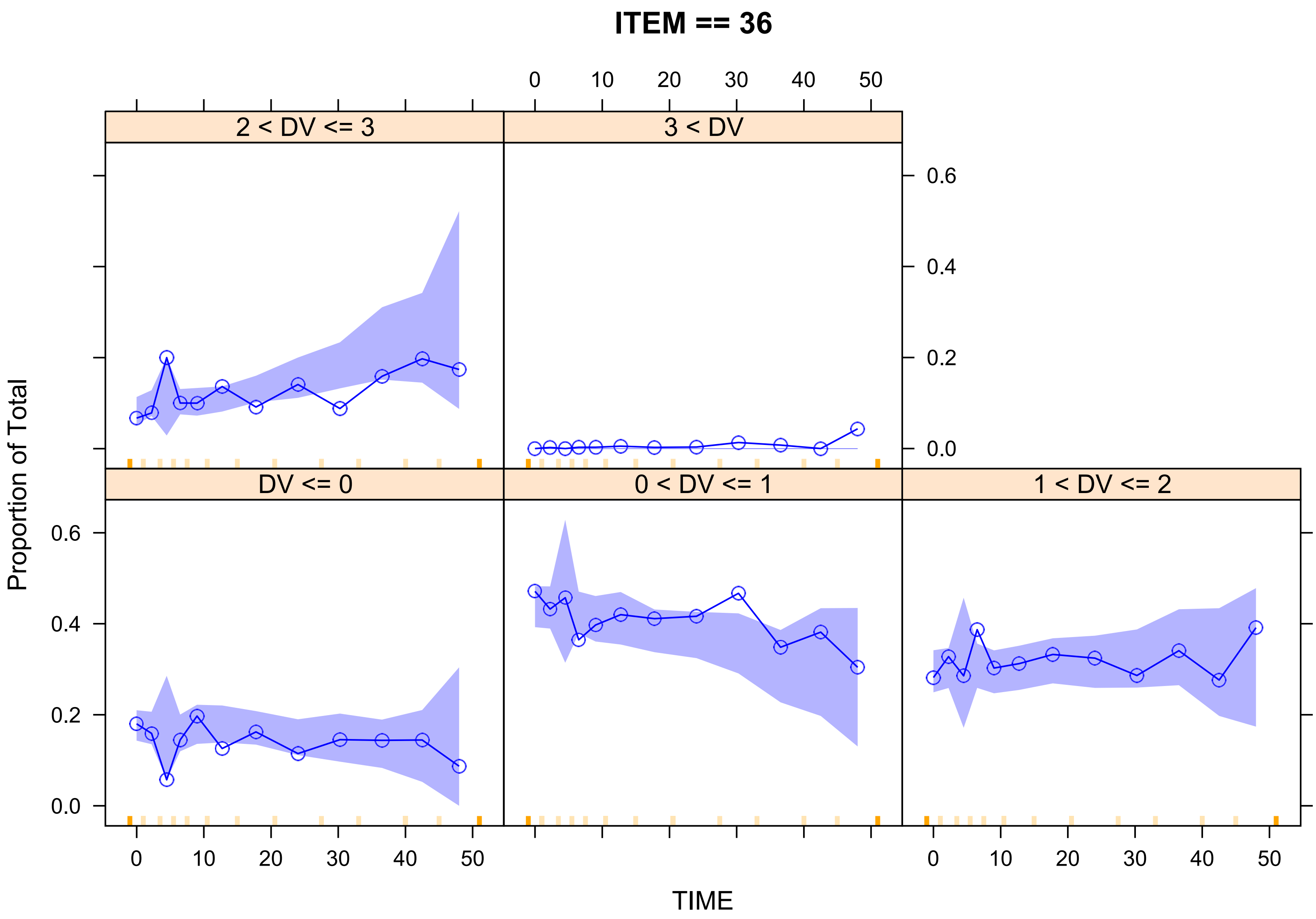

ITEM == 36
2 < DV <= 3
3 < DV
DV <= 0
0 < DV <= 1
1 < DV <= 2
Proportion of Total
TIME
0.0
0.2
0.4
0.6
0
10
20
30
40
50

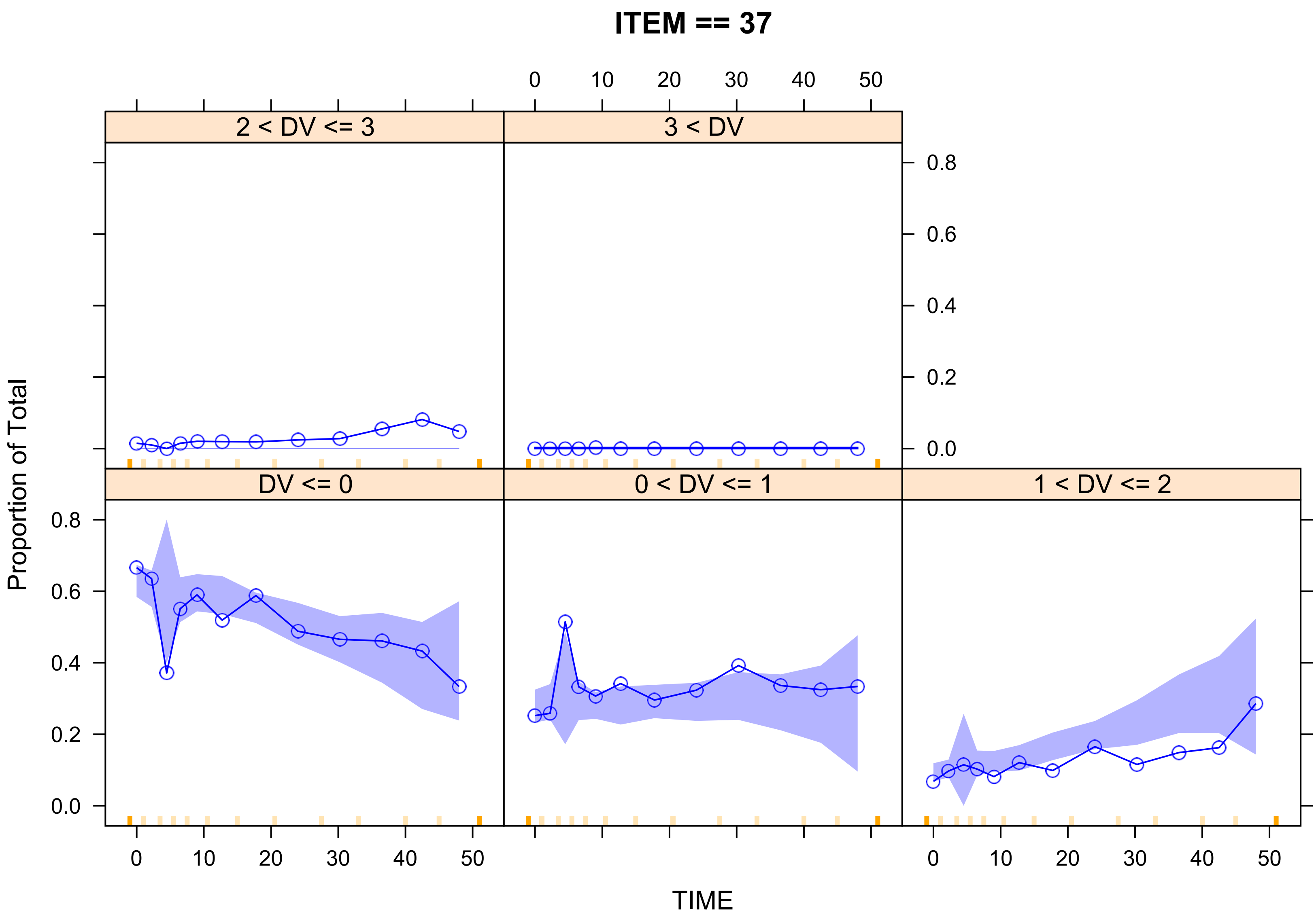

ITEM == 37
2 < DV <= 3
3 < DV
DV <= 0
0 < DV <= 1
1 < DV <= 2
Proportion of Total
TIME
0.0
0.2
0.4
0.6
0.8
0
10
20
30
40
50

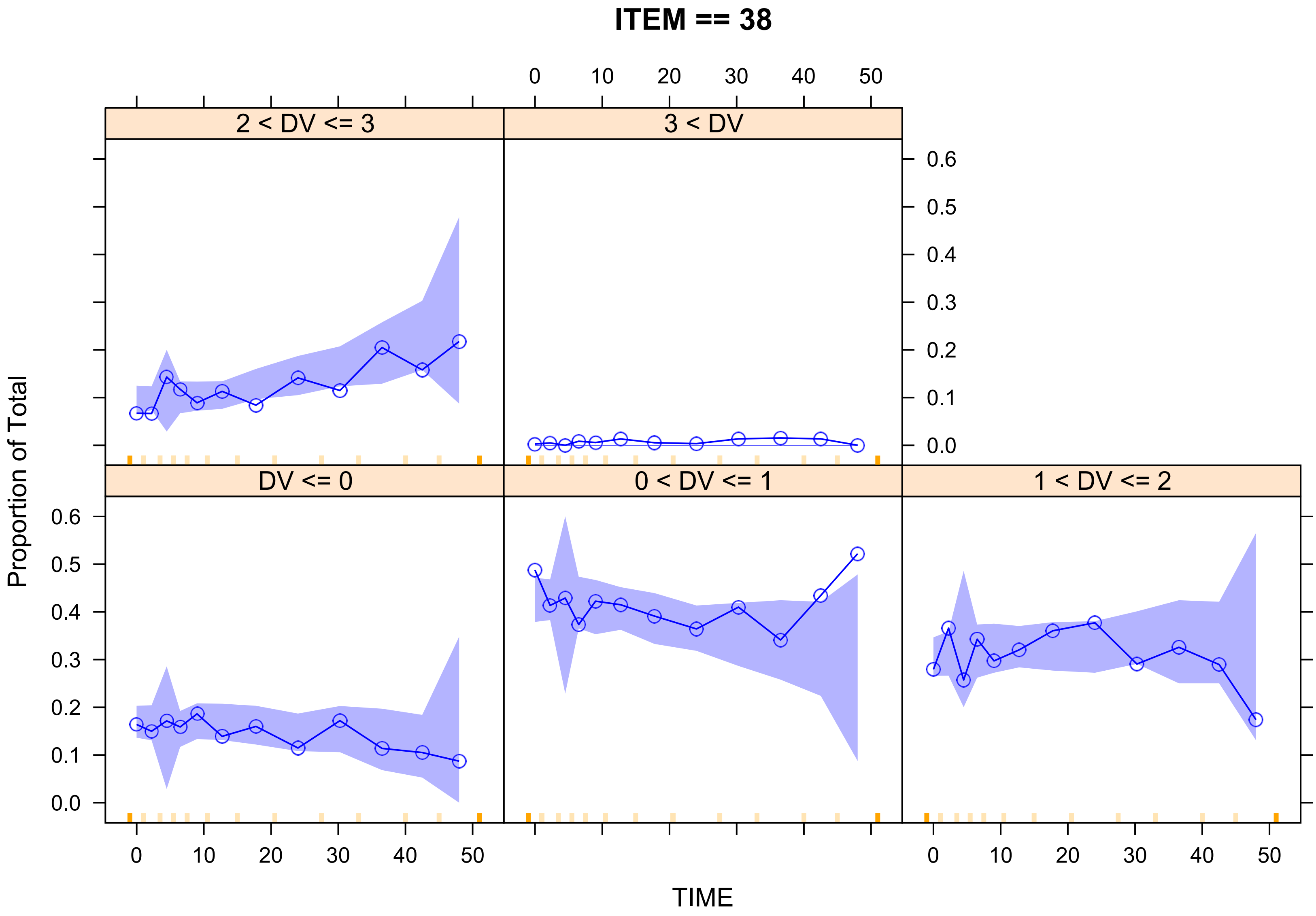

ITEM == 38
2 < DV <= 3
3 < DV
DV <= 0
0 < DV <= 1
1 < DV <= 2
Proportion of Total
TIME
0.0
0.1
0.2
0.3
0.4
0.5
0.6
0
10
20
30
40
50

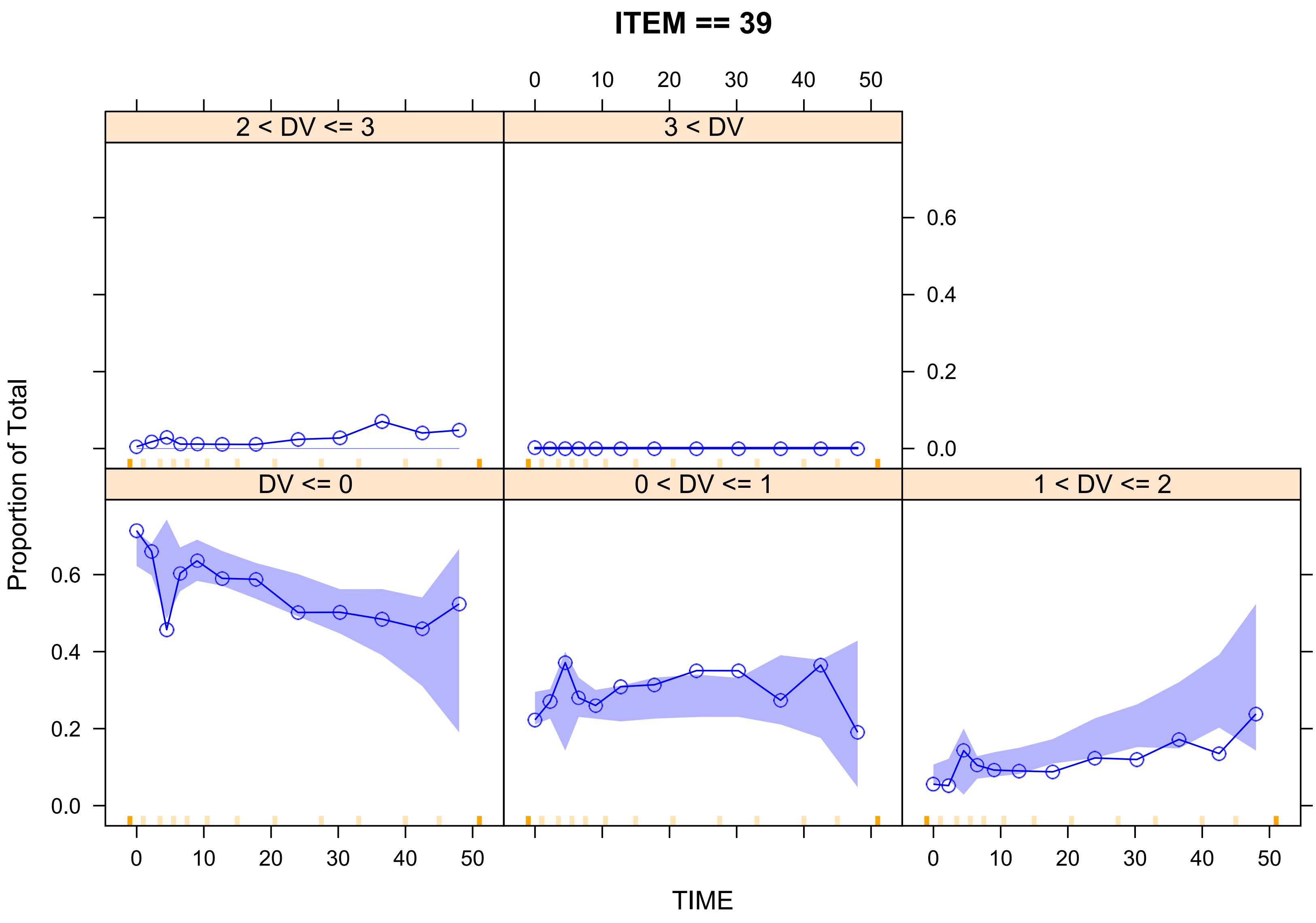
ITEM == 39
2 < DV <= 3
3 < DV
DV <= 0
0 < DV <= 1
1 < DV <= 2
Proportion of Total
TIME
0.0
0.2
0.4
0.6
0
10
20
30
40
50

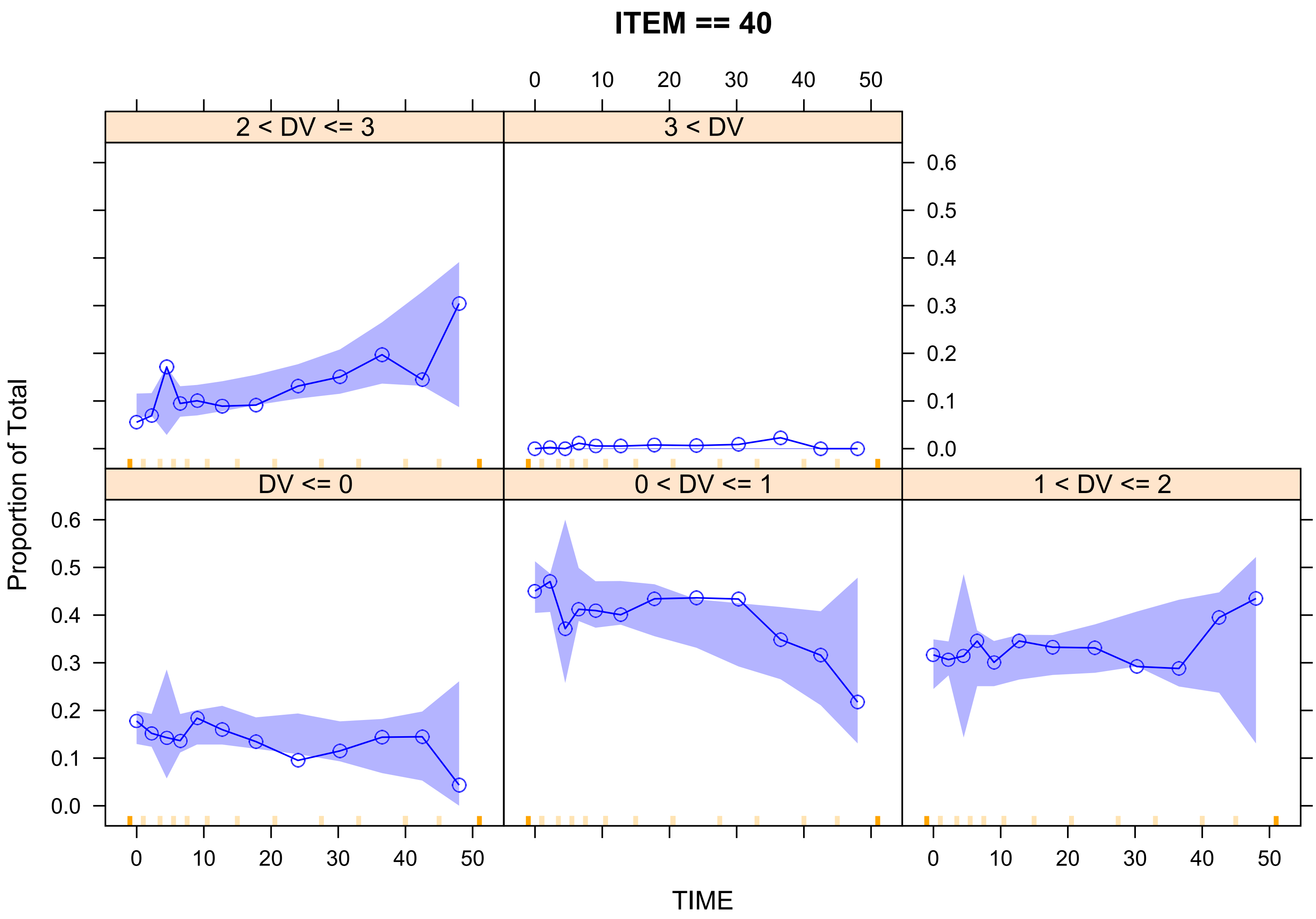
ITEM == 40
2 < DV <= 3
3 < DV
DV <= 0
0 < DV <= 1
1 < DV <= 2
Proportion of Total
TIME
0.0
0.1
0.2
0.3
0.4
0.5
0.6
0
10
20
30
40
50

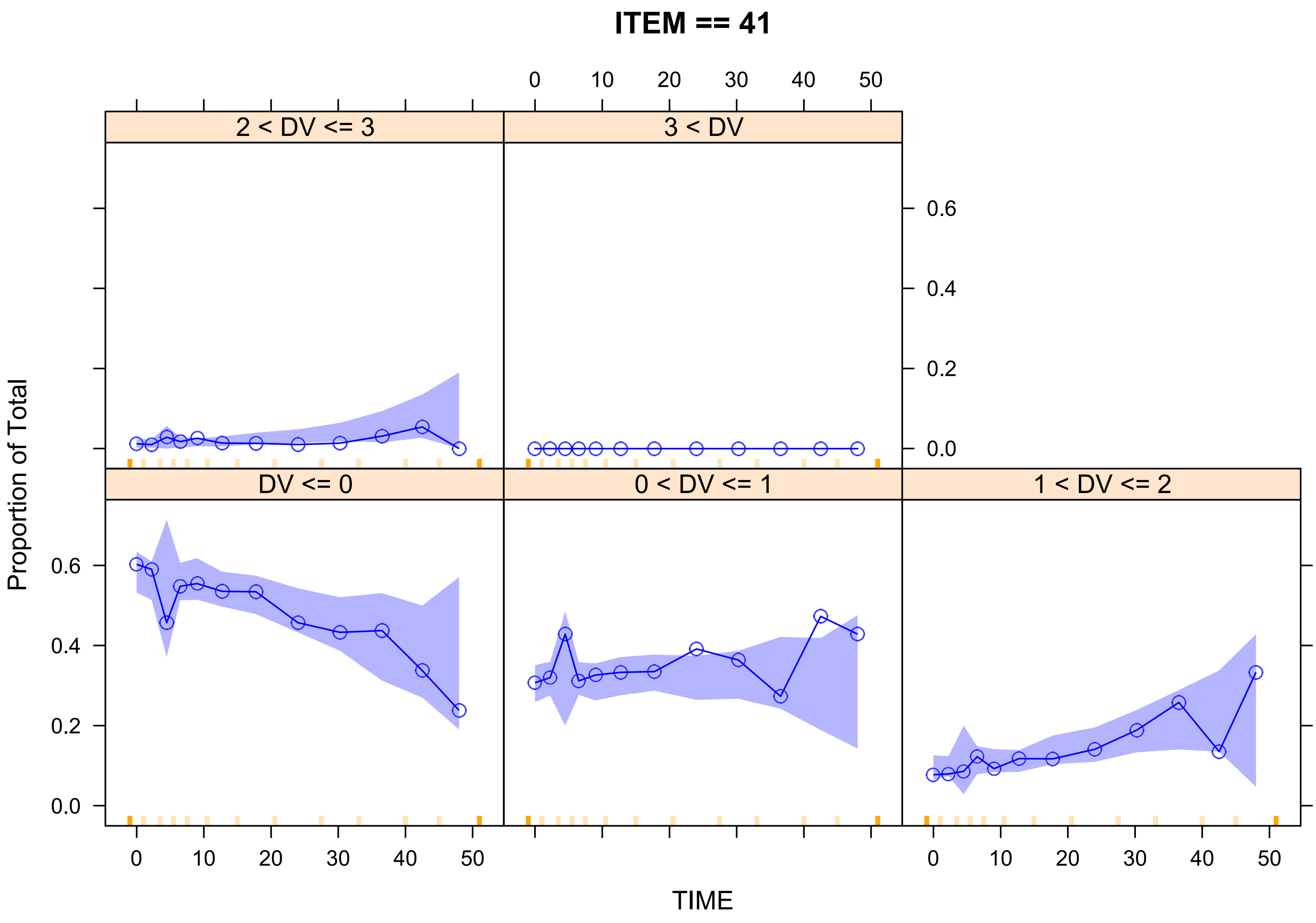

ITEM == 41
2 < DV <= 3
3 < DV
DV <= 0
0 < DV <= 1
1 < DV <= 2
Proportion of Total
TIME
0.0
0.2
0.4
0.6
0
10
20
30
40
50

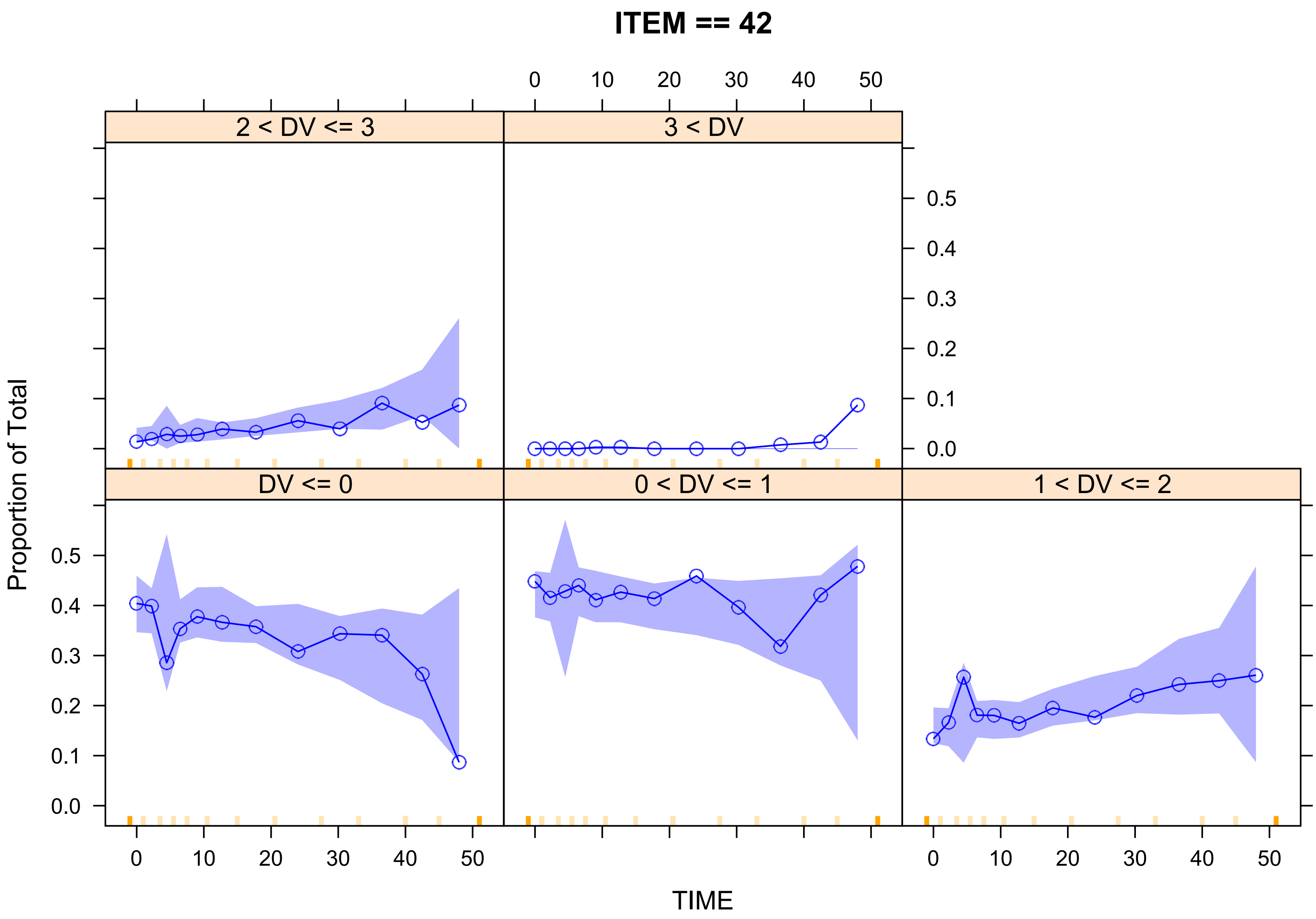
ITEM == 42
2 < DV <= 3
3 < DV
DV <= 0
0 < DV <= 1
1 < DV <= 2
Proportion of Total
TIME
0.0
0.1
0.2
0.3
0.4
0.5
0
10
20
30
40
50

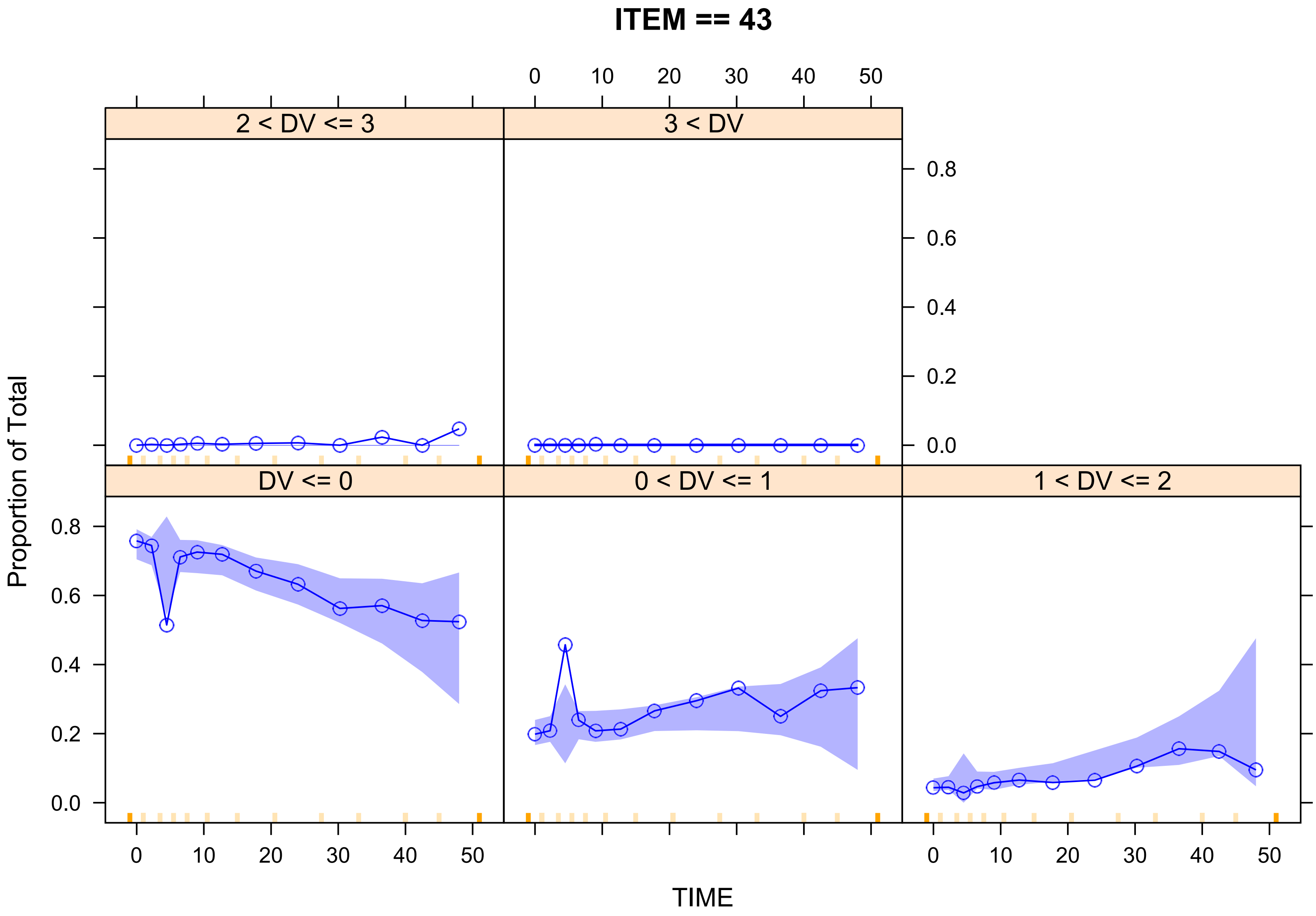

ITEM == 43
2 < DV <= 3
3 < DV
DV <= 0
0 < DV <= 1
1 < DV <= 2
Proportion of Total
TIME
0
10
20
30
40
50
0.0
0.2
0.4
0.6
0.8

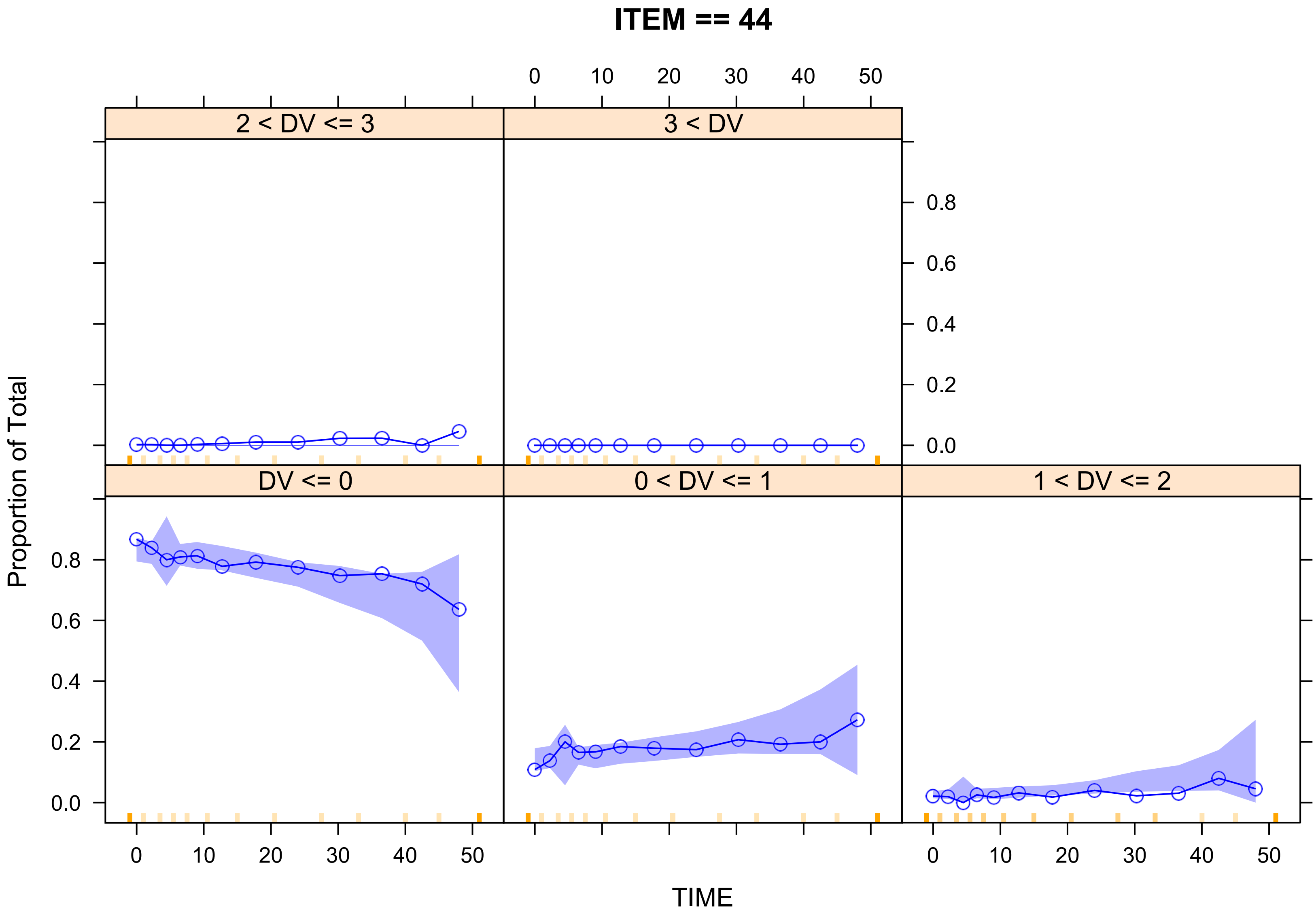
ITEM == 44
2 < DV <= 3
3 < DV
DV <= 0
0 < DV <= 1
1 < DV <= 2
Proportion of Total
TIME
0.0
0.2
0.4
0.6
0.8
0
10
20
30
40
50

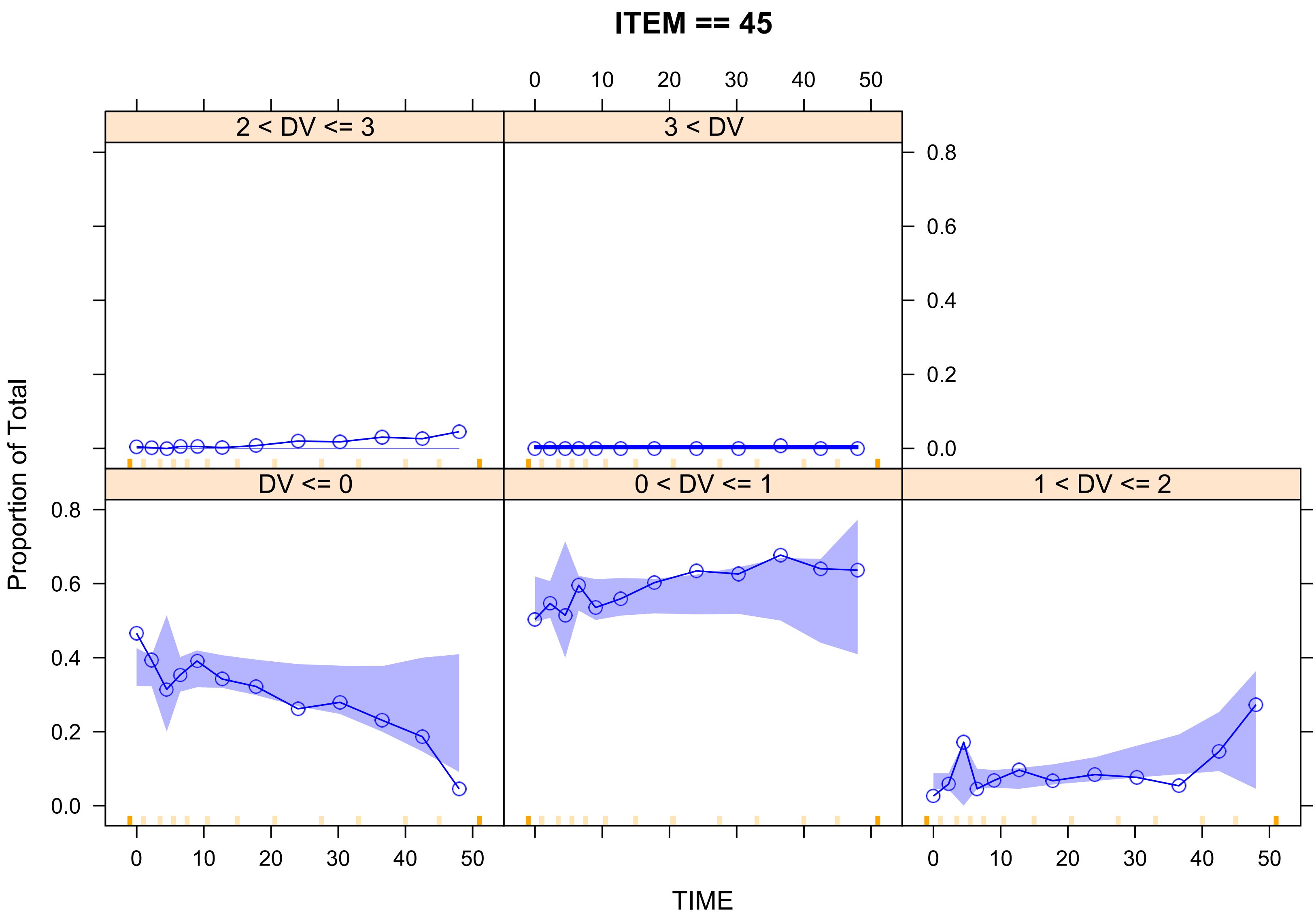

ITEM == 45
2 < DV <= 3
3 < DV
DV <= 0
0 < DV <= 1
1 < DV <= 2
Proportion of Total
TIME
0.0
0.2
0.4
0.6
0.8
0
10
20
30
40
50

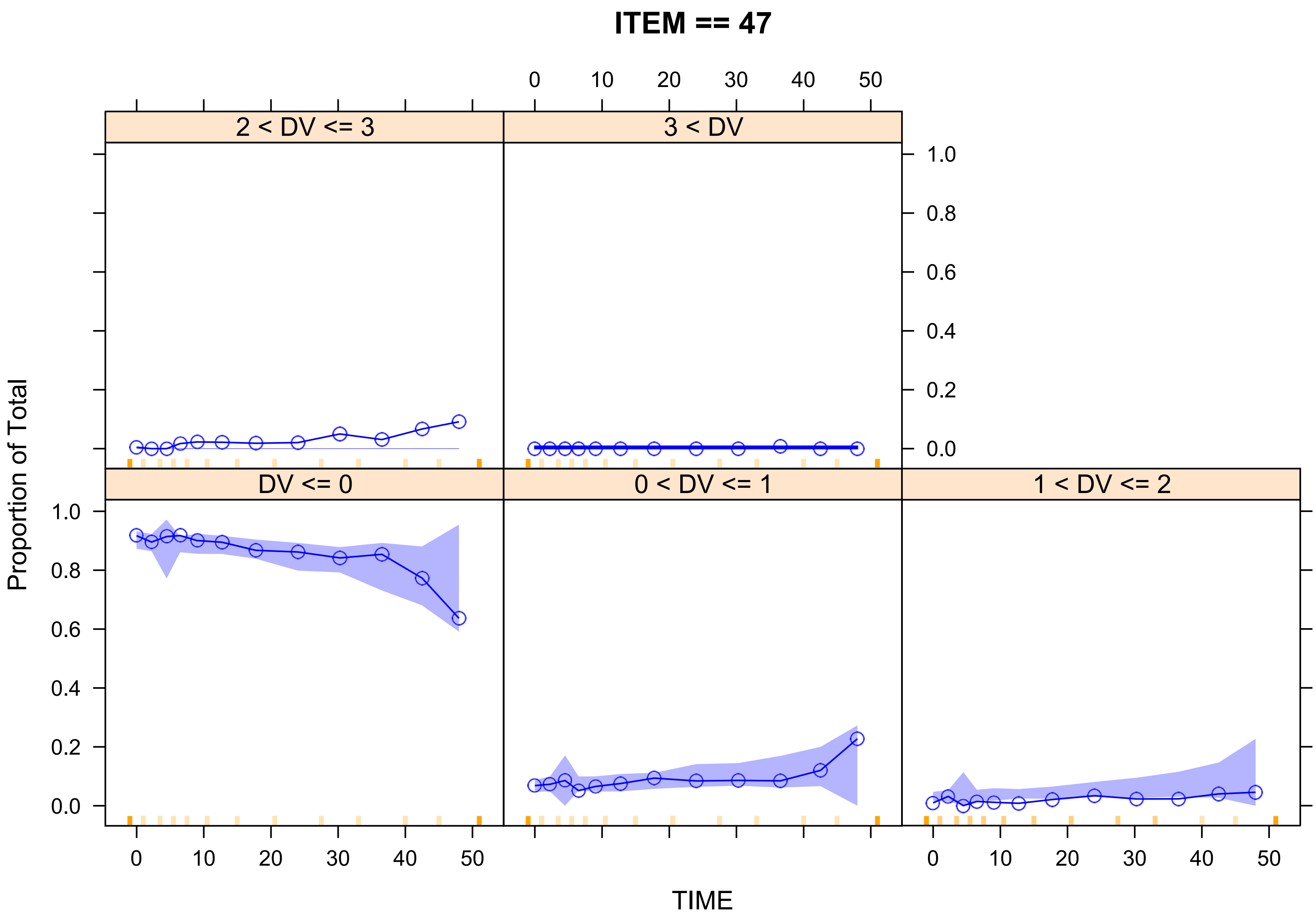

ITEM == 47
2 < DV <= 3
3 < DV
DV <= 0
0 < DV <= 1
1 < DV <= 2
Proportion of Total
TIME
0
10
20
30
40
50
0.0
0.2
0.4
0.6
0.8
1.0

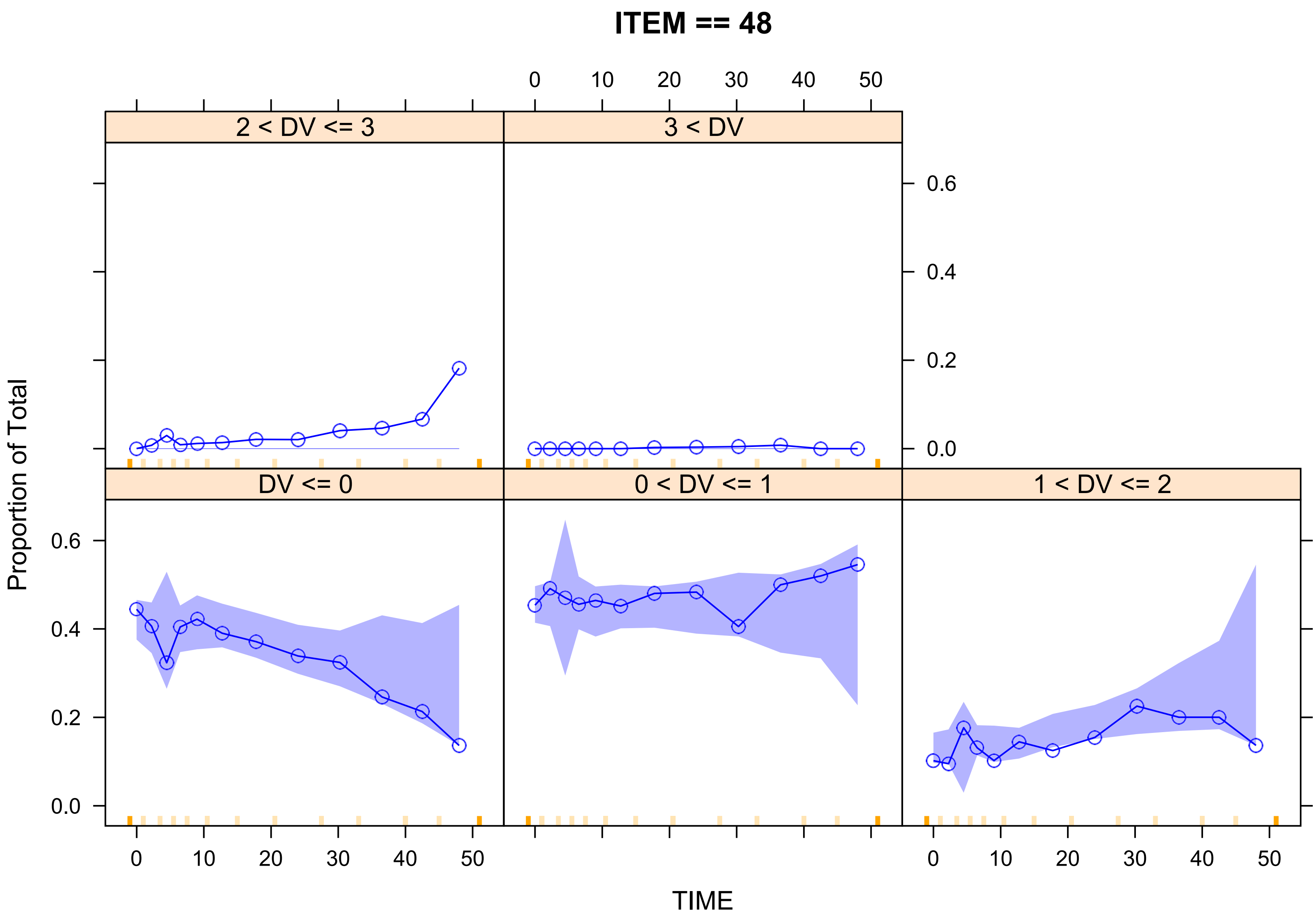

ITEM == 48
2 < DV <= 3
3 < DV
DV <= 0
0 < DV <= 1
1 < DV <= 2
Proportion of Total
TIME
0.0
0.2
0.4
0.6
0
10
20
30
40
50

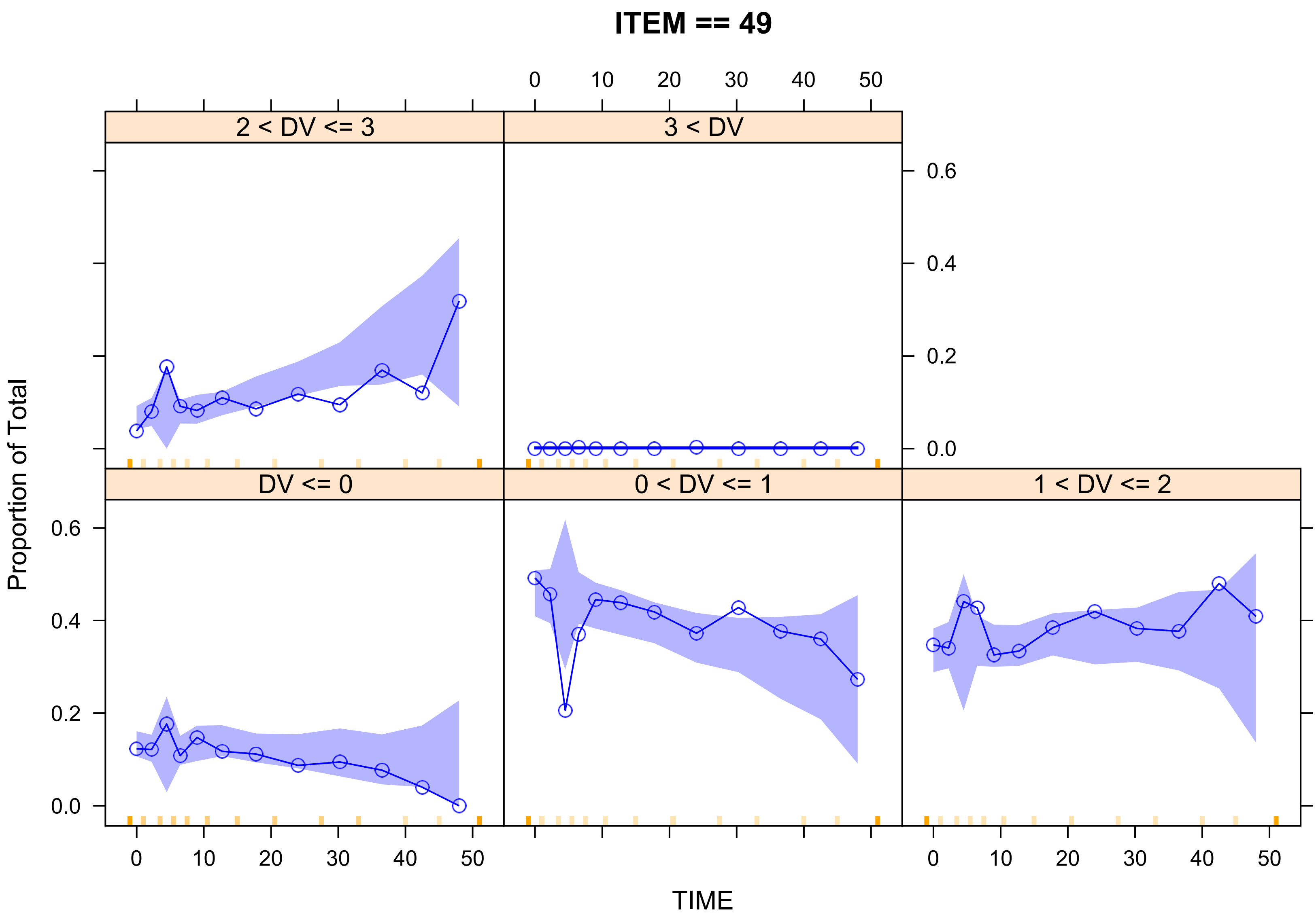

ITEM == 49
2 < DV <= 3
3 < DV
DV <= 0
0 < DV <= 1
1 < DV <= 2
Proportion of Total
TIME
0.0
0.2
0.4
0.6
0
10
20
30
40
50

Figure S2 . Item Characteristic Curves (ICC) for all motor score items in the 100% (all items) scenario. Black dots indicate the observed scores. Panel with “1” (left most column) shows values with scores 0 and ≥1; panel with “2” (second column from left) shows values with scores 1 and ≥ 2; panel with “3” (third column from left) shows values with scores 2 and ≥3; and panel with “4” (right most column) shows values with scores 3 and ≥ 4. The ICC curves from the IRT model fit (dark red line) is compared to the fit of a generalized additive model (GAM) with cross-validated cubic spline as a smoothing function (blue line and the associated 95% confidence interval is shown in grey)

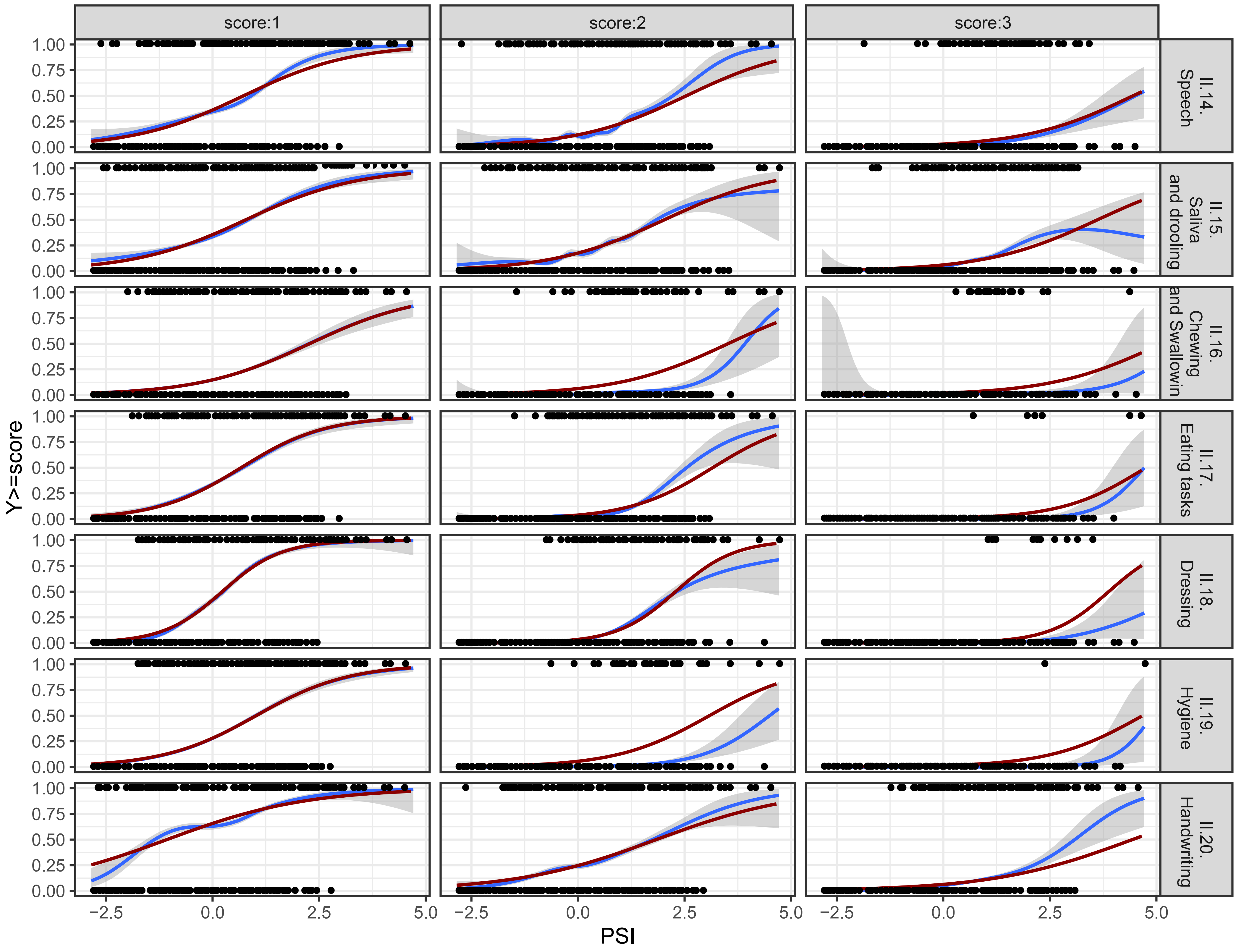
score:1
score:2
score:3
II.14. Speech
II.15. Saliva and drooling
II.16. Chewing and Swallowin
II.17. Eating tasks
II.18. Dressing
II.19. Hygiene
II.20. Handwriting
Y>=score
PSI
1.00
0.75
0.50
0.25
0.00
−2.5
0.0
2.5
5.0

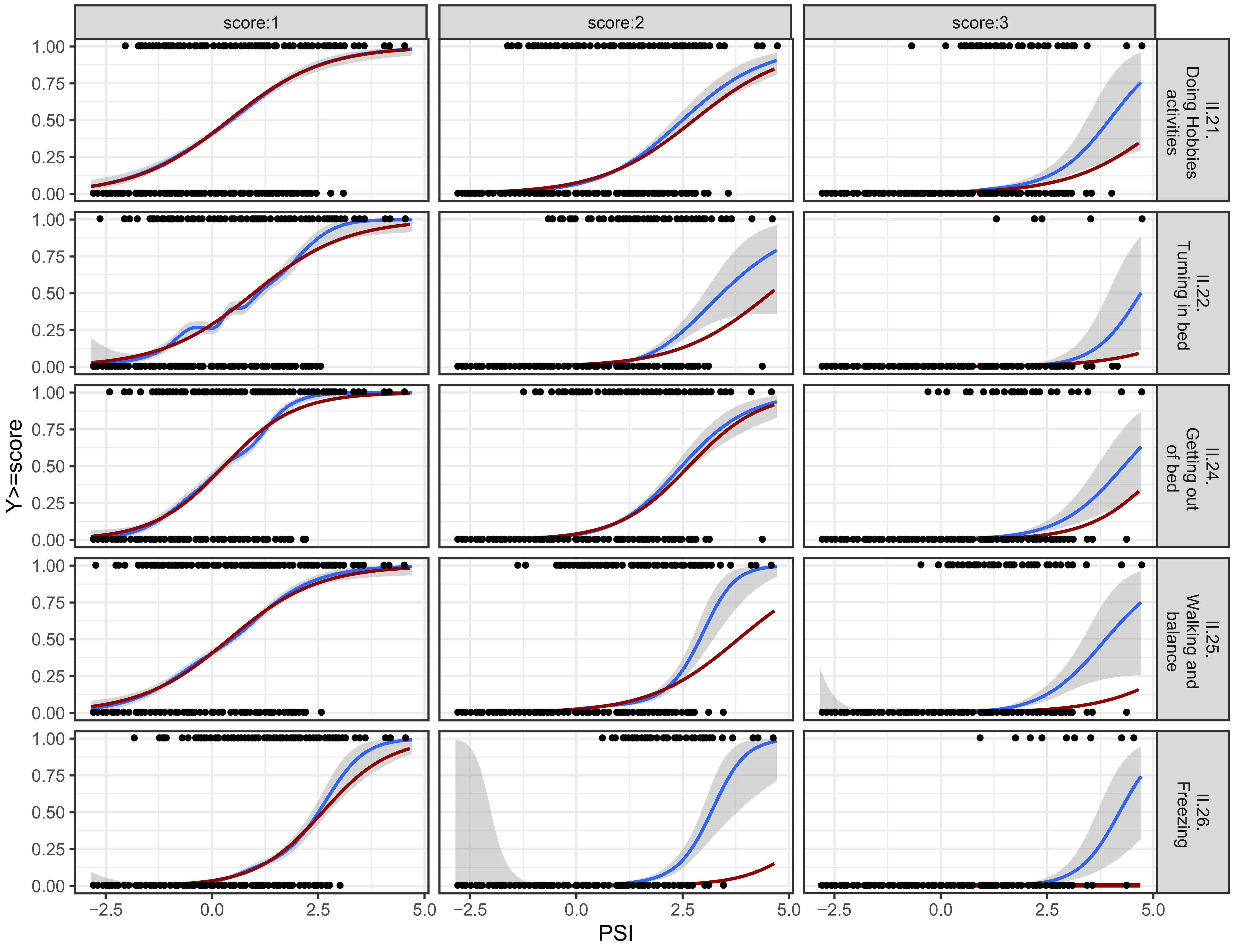

score:1
score:2
score:3
II.21. Doing Hobbies activities
II.22. Turning in bed
II.24. Getting out of bed
II.25. Walking and balance
II.26. Freezing
Y>=score
PSI
1.00
0.75
0.50
0.25
0.00
−2.5
0.0
2.5
5.0

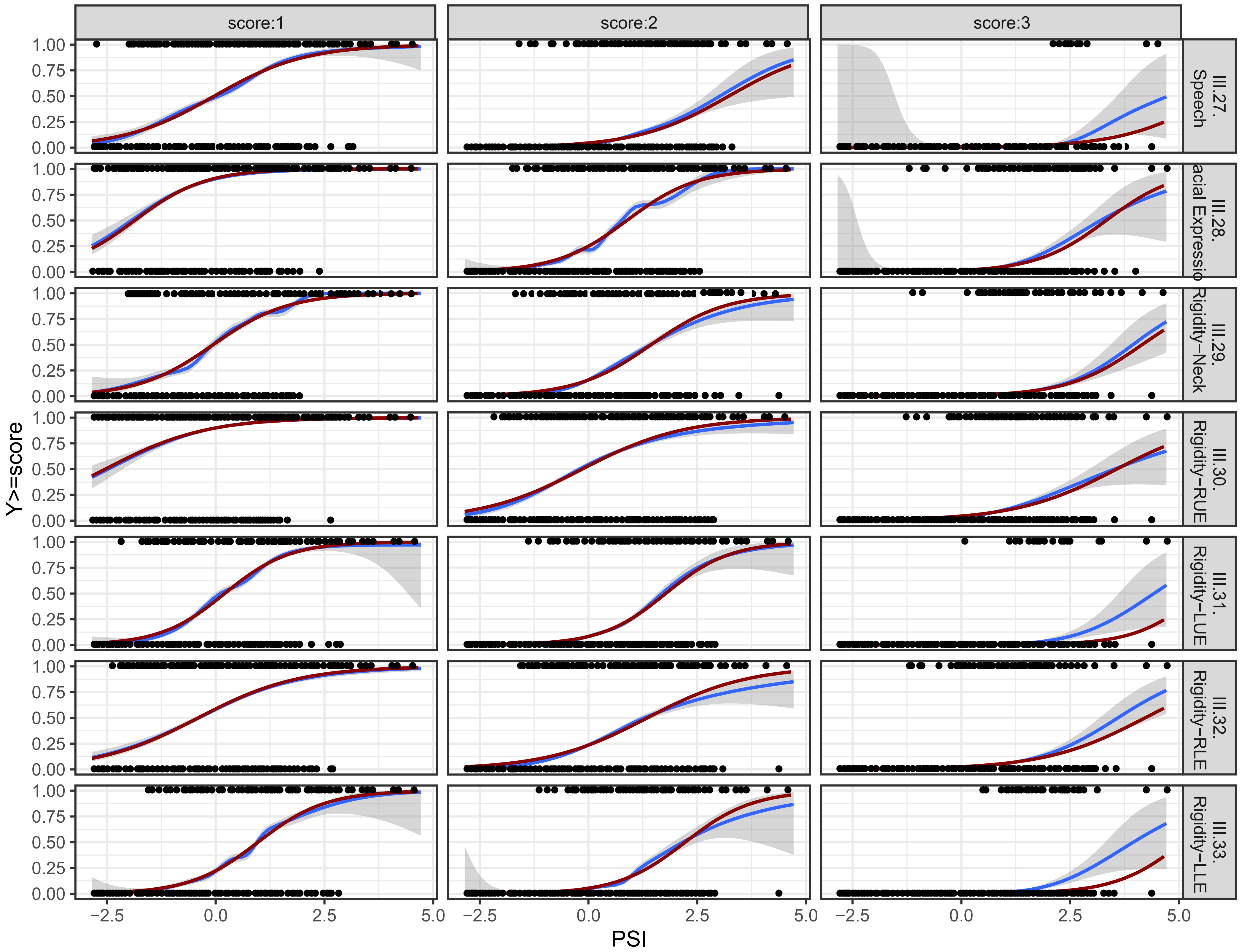

score:1
score:2
score:3
III.27. Speech
III.28. acial Expressio
III.29. Rigidity−Neck
III.30. Rigidity−RUE
III.31. Rigidity−LUE
III.32. Rigidity−RLE
III.33. Rigidity−LLE
Y>=score
PSI
1.00
0.75
0.50
0.25
0.00
−2.5
0.0
2.5
5.0

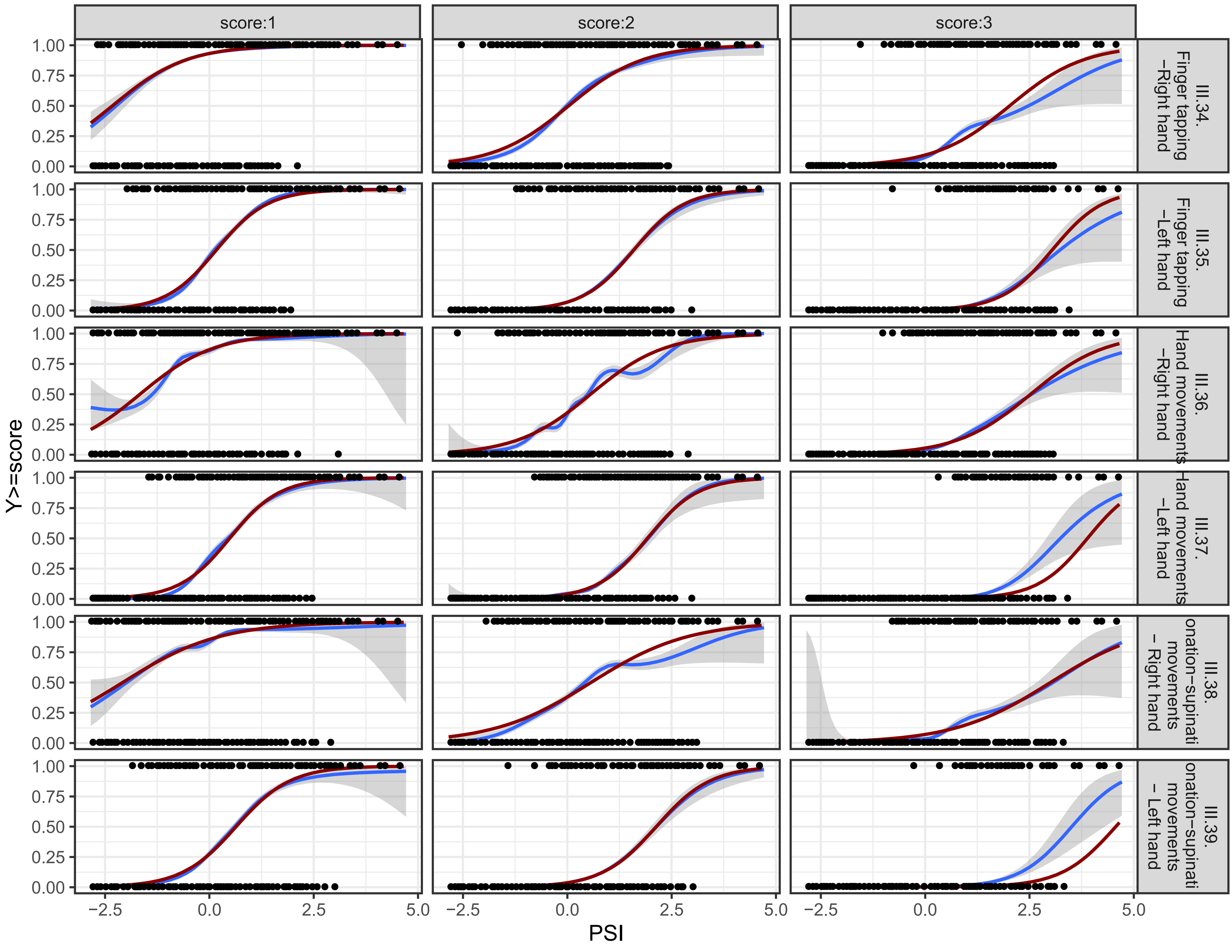

score:1
score:2
score:3
III.34. Finger tapping −Right hand
III.35. Finger tapping −Left hand
III.36. Hand movements −Right hand
III.37. Hand movements −Left hand
III.38. onation−supinati movements − Right hand
III.39. onation−supinati movements − Left hand
Y>=score
PSI
1.00
0.75
0.50
0.25
0.00
−2.5
0.0
2.5
5.0

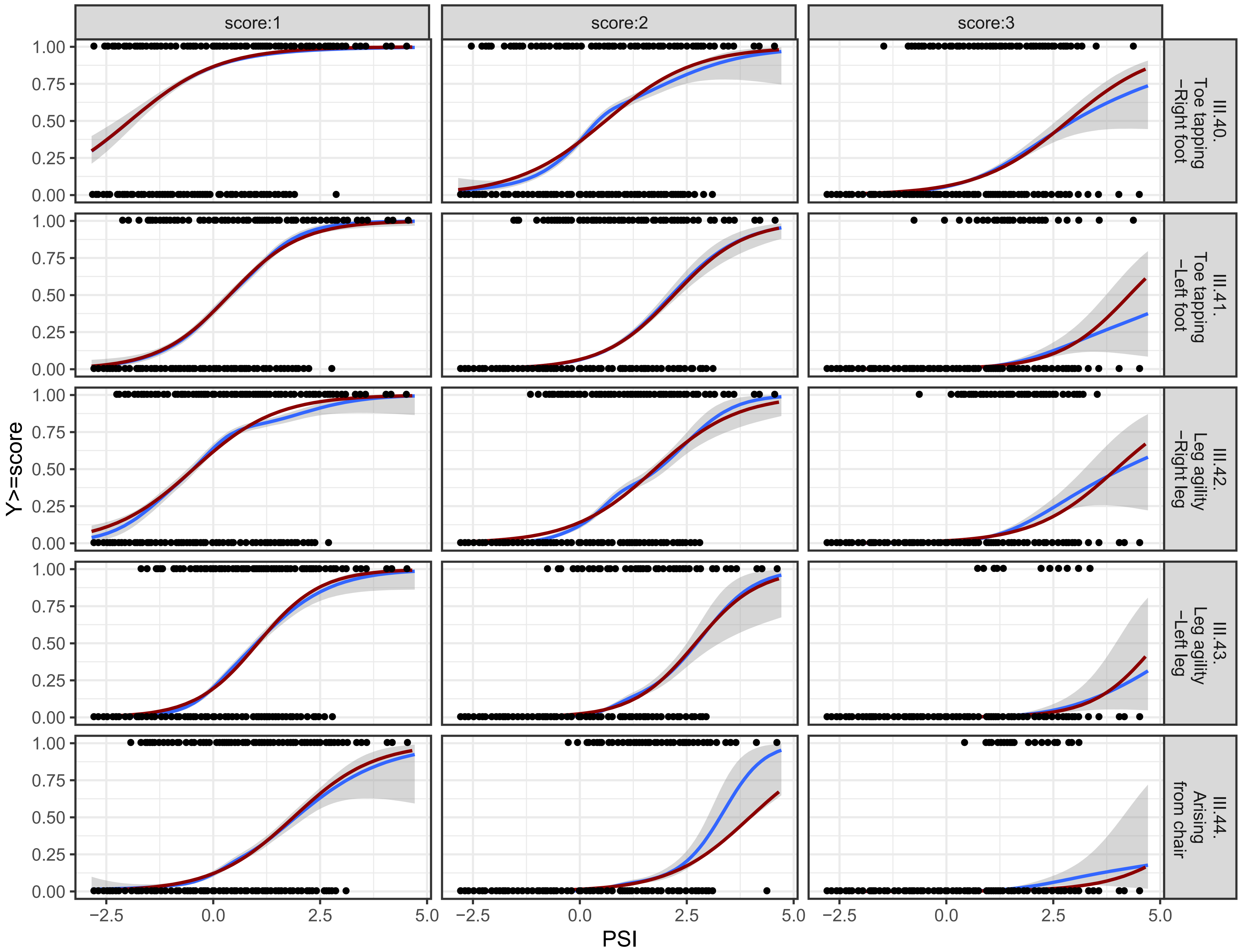

score:1
score:2
score:3
III.40. Toe tapping −Right foot
III.41. Toe tapping −Left foot
III.42. Leg agility −Right leg
III.43. Leg agility −Left leg
III.44. Arising from chair
Y>=score
PSI
0.00
0.25
0.50
0.75
1.00
−2.5
0.0
2.5
5.0

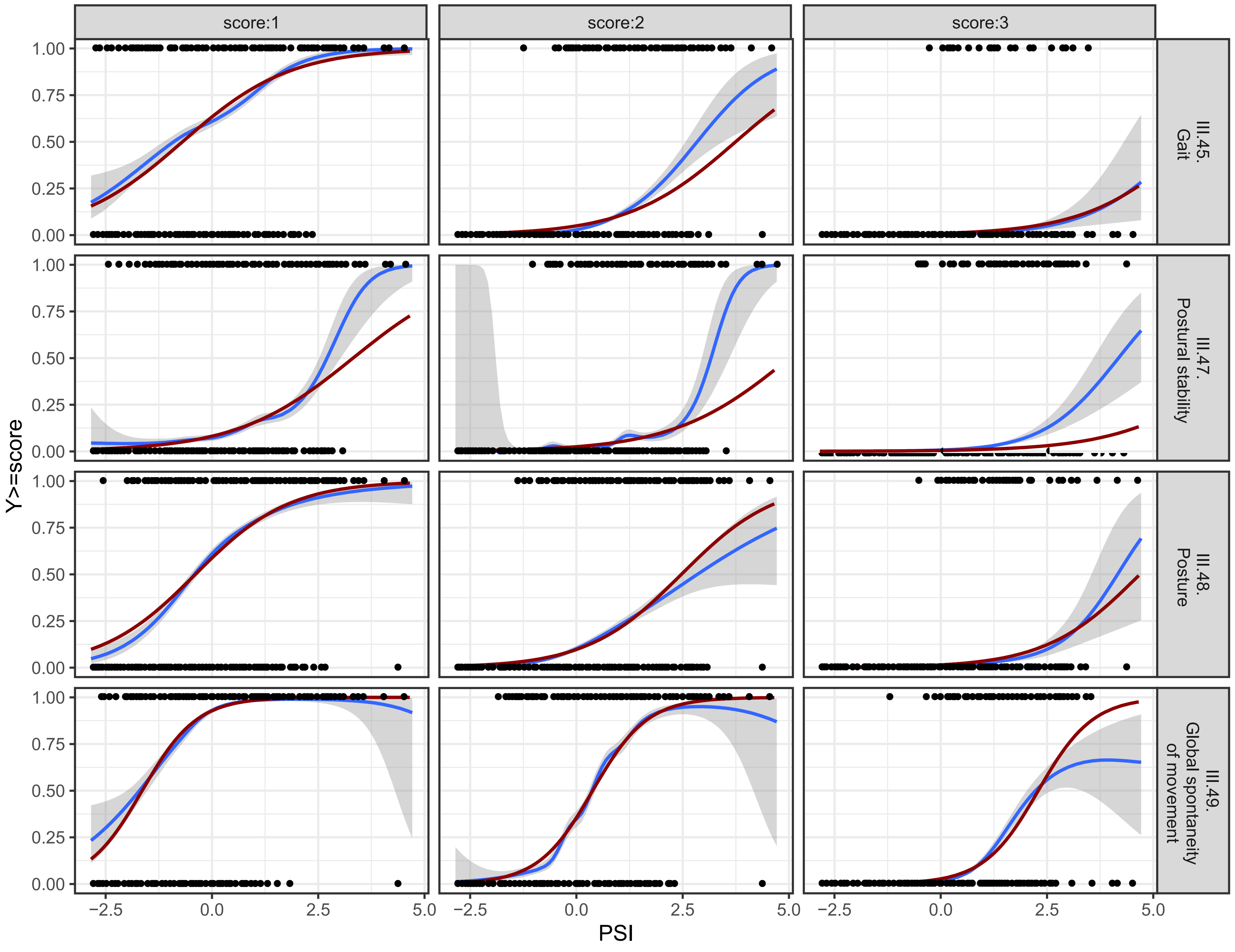

score:1
score:2
score:3
III.45. Gait
III.47. Postural stability
III.48. Posture
III.49. Global spontaneity of movement
Y>=score
PSI
1.00
0.75
0.50
0.25
0.00
−2.5
0.0
2.5
5.0
