# Supplementary material for: Performance of longitudinal item response theory models in shortened or partial assessments
Source: J Pharmacokinet Pharmacodyn. 2020 Jul 2;47(5):461–71. doi: 10.1007/s10928-020-09697-x (PMC7520414; doi:10.1007/s10928-020-09697-x)
Supplement: Supplementary file 3 — Supplementary file3 (DOCX 401 kb) [file 10928_2020_9697_MOESM3_ESM.docx]

**Supplementary Materials 3**

Journal of Pharmacokinetics and Pharmacodynamics

Title: Performance of Longitudinal Item Response Theory Models in Shortened Assessments

Authors: Leticia Arrington^1,2,^ Sebastian Ueckert^1^, Malidi Ahamadi^2*^, Sreeraj Macha^2*^ and Mats O. Karlsson^1,3^

1 Department of Pharmaceutical Biosciences, Uppsala University, P.O. Box 591, 751 24 Uppsala, Sweden

2Merck & Co. Inc. Kenilworth, NJ, USA

3Corresponding author: email Mats.Karlsson@farmbio.uu.se and phone +46 184 714 105

*Affiliation at time of work

Simulated Data Item Level Efficiency Curves

**Fig. S3 Simulated Data Item Level Efficiency for MDS-UPDRS motor items versus Disability (Removal of Least informative Items first). Vertical Lines indicate the disability range for 95% of the reference population**

**Fig. S4**  **Simulated Data Item Level Efficiency for MDS-UPDRS motor items versus Disability (Removal of Most informative Items first). Vertical Lines indicate the disability range for 95% of the reference population**
